# A systematic review of the relation between ten potential occupational sensitizing exposures and asthma

**DOI:** 10.5271/sjweh.4214

**Published:** 2025-05-01

**Authors:** Annett Dalbøge, Henrik Albert Kolstad, Alexander Jahn, Charlotte Suppli Ulrik, David Lee Sherson, Harald William Meyer, Niels Ebbehøj, Torben Sigsgaard, Xaver Baur, Vivi Schlünssen

**Affiliations:** 1 Department of Occupational Medicine, Danish Ramazzini Centre, Aarhus University Hospital, Aarhus, Denmark.; 2 Department of Public Health, Environment, Occupation and Health, Danish Ramazzini Centre, Aarhus University, Aarhus, Denmark.; 3 Department of Respiratory Medicine, Hvidovre University Hospital, Hvidovre, Denmark.; 4 Department of Occupational & Environmental Medicine, Odense University Hospital, Odense, Denmark.; 5 Department of Pulmonary Medicine, Odense University Hospital, Odense, Denmark.; 6 Department of Occupational & Environmental Medicine, Bispebjerg & Frederiksberg Hospital, University of Copenhagen, Copenhagen, Denmark.; 7 Institut für Arbeitsmedizin, Charité Universitätsmedizin Berlin, Berlin, Germany.

**Keywords:** Allergen, allergy, lung disease, respiratory symptom, work

## Abstract

**Objective:**

The aim of this systematic review was to identify, evaluate, and synthesize the relation between 10 potential occupational sensitizing exposure groups and asthma.

**Methods:**

A systematic literature search was conducted in three databases for peer-reviewed articles published between July 2011 and March 2023. Exposures included ten potential occupational sensitizing exposure groups (amines, anhydrides, biocides [eg, pesticides], crustaceans, enzymes, mammals, metals, “mold, fungi and yeast”, molluscs, and other chemicals [eg, cleaning agents]) classified as having no or limited evidence of a causal relation with asthma in our previous overview of systematic reviews. We included observational and case studies. Study selection, data extraction, risk of bias assessment, and evidence level evaluation were conducted independently by two reviewers, who also upgraded or downgraded the level of evidence found in our overview.

**Results:**

This review included 55 articles. The overall confidence in study results was rated high in 8, moderate in 18, and low in 29 studies. No new studies were found for molluscs. For the remaining exposures, we upgraded main groups of crustaceans and enzymes to moderate evidence, mammals and metals to limited/contradictory, and amines and biocides to very limited/contradictory. For subgroups/specific exposures, pesticides, cleaning agents – such as chloramine and disinfection products – and an unspecified group of other chemicals, specifically acrylates and epoxy, were upgraded to moderate.

**Conclusion:**

New occupational sensitizing exposures with moderate evidence include crustaceans, enzymes, pesticides, cleaning agents such as chloramine and disinfection products, and chemicals such as acrylates and epoxy.

Asthma is a common chronic disease among children and adults, with symptoms such as coughing, shortness of breath, and wheezing. The disease is characterized by airway inflammation and hyper-responsiveness, leading to variable degree of airway re-modelling (1). Based on recent multi-country data, the global prevalence of asthma is 4.4% (0.9–29.0%) (2). It is estimated that 15% of adult asthma is related to occupational exposures (3, 4).

Asthma caused by occupational exposure can be due to specific immunological mechanisms, either driven by IgE-mediated sensitization or other less well characterized immunological mechanisms (5, 6). The relation between potential occupational sensitizing exposures and asthma has been reported in hundreds of studies with a wide range of exposures. In addition, numerous reviews have been published, with the vast majority being scoping reviews or discussion papers. In a comprehensive systematic review of 372 potential occupational sensitizing exposures, Baur et al (7) found strong evidence of a causal relation for exposure to various laboratory animals, moderate evidence for 35 exposures, and limited or no evidence for the remaining exposures. In Baur et al's review, potential occupational sensitizing exposures were divided into main groups (eg, mammals) and subgroups/specific exposures (eg, mice). We recently conducted an overview of systematic reviews including 1189 studies of almost 500 potential occupational sensitizing exposures (8). In this overview, we found strong evidence of a causal relation for main groups of wood dusts and moderate evidence for main groups of mites and fish. For subgroups/specific exposures, we found strong evidence for exposure to laboratory animals and moderate evidence for 55 subgroups/specific exposures. For the remaining exposures, limited/contradictory or no evidence was found.

New studies on the relation between potential occupational sensitizing exposures and asthma have recently been published. The aim of this systematic review was to identify, appraise, and synthesize the relation between ten potential occupational sensitizing exposure groups and asthma. These exposure groups (amines, anhydrides, biocides, crustaceans, enzymes, mammals, metals, “mold, fungi and yeast”, molluscs, and other chemicals) were found to have no or limited/contradictory evidence of a causal relation with asthma in our overview of systematic review (8).

## Methods

### Protocol and registration

This study was the second of two studies on the relation between potential occupational sensitizing exposures and asthma requested by the National Board of Industrial Injuries and the Occupational Diseases Committee in Denmark (9). Our study protocol was registered in PROSPERO (CRD42017057014). We followed specific guidelines for preparation and quality approval provided by the Preferred Reporting Items for Systematic Reviews and Meta-Analyses (PRISMA) statement (10). To ensure the methodological quality of our systematic review, we also followed guidelines provided by Assessing the Methodological Quality of Systematic Reviews (AMSTAR 2).

### Literature search, eligible criteria and exclusion of studies

We constructed a PECOS (Population, Exposure, Comparison, Outcome, Study design) for study eligibility (supplementary material, www.sjweh.fi/article/4214, appendix 1). The population included persons of or older than working age, the exposure comprised ten specified potential occupational sensitizing exposure groups (see below), we compared different exposure levels, and included studies where outcome was defined as asthma. Eligible study designs included epidemiological (observational eg, cross-sectional, case–control, and cohort studies) and clinical studies (case studies eg, case reports and case series). Clinical studies were only included if outcome assessment was based on self-reported symptoms in combination with objective measurements. The ten potential occupational sensitizing exposure groups were selected among groups of sensitizing exposures with no scientific to limited evidence of a causal relation found in our overview of systematic reviews (8). We prioritized frequent potential occupational sensitizing exposures, suspected low molecular weight exposures, and exposures which are not considered well known causes of asthma (eg, flour). The ten selected sensitizing exposure groups included: amines, anhydrides (not Phthalic anhydride), biocides, crustaceans (not lobsters and snow crabs), enzymes (not a-amylase from *Aspergillus oryzae*, detergent enzymes, Papain, Phytase from *Aspergillus niger*, various enzymes from *Bacillus subtilis* (alcalase, protease, maxatase, maxapem, esperase, cellulase, a-amylase, lipase, subtilisin), mammals (not cows, rats), metals (not Platinum salts), mold, fungi and yeast (not *Aspergillus, Cladosporium, Penicillium*), molluscs, and other chemicals (not drugs, dyes, biocides, and isocyanates). The exposures in parenthesis were excluded as we found moderate-to-strong evidence of a causal relation for these subgroups/specific exposures in our overview (8). “Other chemicals” was considered a subgroup of chemicals and are not strictly defined but contain highly reactive chemicals like cleaning agents, and an “unspecified group of other chemicals” including acrylates, epoxy resin, and persulfates. To disentangle the effect of specific cleaning agents, we decided to include cleaning agents, even though moderate evidence for unspecific cleaning agents was documented in our overview. To be as comprehensive as possible, we did not exclude studies of exposures, which are considered a mixture of possible sensitizing exposure and other types of exposures eg, metal fluid.

In collaboration with a librarian, the literature search was conducted in three databases ie, the National Library of Medicine (MEDLINE/PubMed), Embase, and Web of Science (WoS) (supplementary appendix 2) for peer-reviewed articles published between 1 January 2011 and 29 March 2023; as July 2011 was the date of the literature search in Baurs review (7). In Covidence (www.covidence.org), article duplicates and articles published before July 2011 were excluded. Two reviewers independently excluded articles based on title/abstract screening and full paper reading (supplementary appendix 3). Disagreement was resolved by consensus. Several articles were well known to the review authors, so we did not blind for authorship. We screened the reference lists of all included articles for additional relevant articles.

### Data extraction and risk of bias assessment for each article

For each included article, two reviewers independently extracted study descriptive data comprising author, study design, population, participation rate, exposure definition and assessment, outcome definition and assessment, confounders, and exposure-response analysis. We also extracted information on the relation between potential occupational sensitizing exposures and asthma for both main groups (eg, mammals) and subgroups/specific exposures (eg, mice).

Two reviewers independently assessed the methodological quality of each included article using a “risk of bias” tool developed for this study. This tool included 10 items; items 1–9 concerned study design, population, participation rate, exposure specificity, exposure assessment (I–II), outcome assessment, confounders adjusted for, and exposure–response relation, which could be scored high risk of bias (“0”) or low risk of bias (“1”). Item 10 was a subjective rating of the overall confidence in study results based on items 1–9. The overall confidence in study results could be scored “high”, “moderate”, “low” or “critically low” (supplementary appendix 4). Disagreement on item quality was resolved by consensus. For two randomly selected articles (ie, an observational study and a case report), the reviewers pilot-tested the risk of bias tool, discussed disagreements, and reached a consensus.

### Evidence across articles

Meta-analyses could not be conducted due to too few and/or significant heterogeneity in methodology (eg, exposure and outcome definition and assessment) among the included studies. Therefore, we chose to present a synthesis of the included studies in accordance with synthesis without meta-analysis (SWiM) reporting guidelines (11).

Two reviewers independently up- or downgraded the level of evidence for main and subgroups/specific exposures (8) using a modification of the Royal College of General Practitioners' three-star system of the British Occupational Health Research Foundation (supplementary appendix 5) (7, 12).

## Results

### Literature search and exclusion of studies

**Figure 1:**
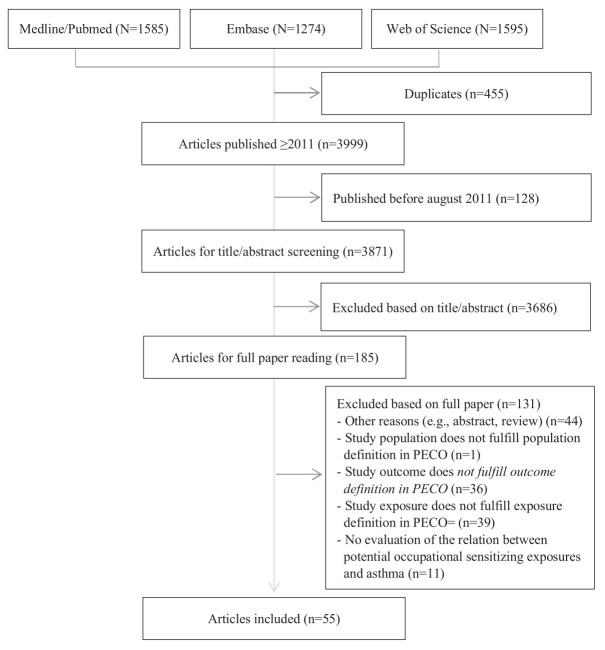
Flow chart of the literature search and exclusion of studies.

A flow chart of the literature search and exclusion of articles based on eligibility are presented in figure 1. A total of 4454 articles were identified from the three databases. We excluded 455 duplicates and 128 articles published before July 2011. After title/abstract screening and full paper reading, we excluded additional 3686 and 131 articles, respectively, providing 55 articles fulfilling the criteria for inclusion in the present review. Supplementary appendix 6 lists the 131 excluded articles and the explanation for their exclusion.

### Study characteristic and risk of bias

Study characteristics and risk of bias of the 55 included studies are presented in table 1 and supplementary appendix 7. In total, 11 cohort studies, 3 case–control studies, 13 cross-sectional studies, and 28 case reports or case series were included. The overall confidence in study results was rated high in 8, moderate in 18, and low in 29 studies. Among all 55 studies, the most frequent items scoring “high risk of bias” were assessment of potential information bias, exposure specificity, and objective measurement of variability of lung function. The most frequent items scoring “low risk of bias” were population, participation rate, and confounders adjusted for. The source of funding for each of the included reviews was evaluated but not reported. The reviewers disagreed in 7% of the ratings.

**Table 1:** Study characteristics and risk of bias of the 55 included studies published 2011–2023. [CC=case-control study; CR=case report; CSS=cross-sectional study; FeNO=fractional exhaled nitric oxide; ICD=International Classification of Diseases; Ig=immunoglobulin; IS=induced sputum; JEM=job exposure matrix; PI=participation rate; SIC=specific inhalation challenge; SPT=skin-prick test].

Author	Study design	Population	PR	Exposure			Outcome
				Definition	Assessment		Assessment
Al-Abcha et al (13)	CS	Workers using or manufacturing carbide tools (N=35)	100%	Metals	Self-reported job history (interview) and workplace enforcement inspections		Physician diagnosed asthma based on symptoms and lung function tests
Baur et al (14)	CR	Chemical worker in the production and packaging (N=1)	100%	Enzymes	Self-report and expert assessment		History of work-related asthma symptoms, lung-function test, IgE measurement, SPT
Beach et al (15)	Cohort	Workers with a claim to Workers’ Compensation Board (N=11 486)	83%	Different exposures	Register information on occupational code combined with an expert-based JEM		Physician billing for asthma (ICD-9 493) 12 months before a Workers’ Compensation Board claim without asthma previous years
Bertelsen et al (16)	CR	Worker from a seafood plant (N=1)	100%	Crustaceans	Self-report, expert assessment, SIC		History of work-related asthma symptoms, lung function tests, IS, IgA, IgE, IgM, IgG, IgE
Branicka et al (17)	CR	Oyster mushroom farmer (N=1)	100%	Mushrooms	Self-report and prick-to-prick test with oyster mushroom		Lung function test, IgE measurements, SPT
Brooks et al (18)	CSS	Cleaners, retail/service workers, and bus drivers (N=706)	34-74%	Other chemicals	Self-report		Questionnaire based interview, lung function tests, and SPT
Carder et al (19)	CS	Patients from surveillance schemes (N=779)	100%	Other chemicals	Information recorded by physicians and the recorded occupation and industry		Physician-diagnosed asthma
Cha et al (20)	CSS	Farmers near an oil spill (N=2882)	NS	Pesticide	Interview		Questionnaire: Asthma defined in terms of the subject having ever been diagnosed with the disease by a physician
Dumas et al (21)	CSS	Workers from population-based biobank and volunteers (N=34 015)	88.3%	Different exposures	Longest held job combined with a asthma-specific JEM		Interview by medical personnel: “Do you have asthma now”, asthma confirmed by physician, health status and medication
Dumas et al (22)	Cohort	Nurses from the Nurses’ Health Study II (N=61 539)	52.8%	Other chemicals	Questionnaire and a Job-Task-Exposure matrix		Questionnaire: Self-reported diagnose of asthma and the use of asthma medication
Dumas et al (23)	Cohort	Nurses from the Nurses’ Health Study III (N=17 280)	63%	Other chemicals	Questionnaire		Questionnaire: Self-reported clinician-diagnosed asthma
Fishwick et al (24)	CSS	Pesticide applicators (N=2578)	54%	Other chemicals	Self-reported exposure to pesticides from a questionnaire		Questionnaire: Self-reported physician-diagnosed asthma: “Has a doctor ever told you that you have asthma?”
Ghosh et al (25)	Cohort	National Child Development cohort (N=7406)	NS	Different exposures	Job history (interview) combined with an asthma- specific expert-based JEM		Lung-function test, IgE. Interview: Self-reported adult-onset asthma
Gonzalez et al (26)	CSS	Healthcare workers (N=543)	77%	Other chemicals	Questionnaire, material data sheets, workplace observations		Self-reported physician diagnosed asthma (“Have you ever had asthma” and “Was it confirmed by a doctor”), respiratory symptoms, IgE measurements
Helaskoski et al (27)	CS	Asthma patients from occupational medicine clinic (N=5)	100%	Highly reactive chemicals	Questionnaire, SIC		History of respiratory symptoms, lung-function test, IgE measurements, SPT, open skin testing, patch test
Hougaard et al (28)	CR	Hairdresser (N=1)	100%	Other chemicals	Self-report and expert assessment		History of work-related asthma symptoms, lung-function test, SPT, patch test
Hoy et al (29)	Cohort	School children (N=792)	NS	Different exposures	Self-reported job history and an asthma-specific JEM		Questionnaire: Asthma at the age of 44 defined as “Have you ever in your life suffered from attacks of asthma or wheezy breathing?
Huang et al (30)	CC	Asthma patients from a general hospital. Controls from same residential area (N=1102)	NS	Metals	Interview, urinary measurements		History of asthma symptoms, physician-diagnosed asthma, lung-function test
Huntley et al (31)	CS	Patients with asthma caused by office work (N=47)	100%	Different exposures	Work history using database, SIC		Lung function test, IgE measurements
Jungewelter et al (32)	CR	35-year-old slaughterhouse worker (N=1)	100%	Mammals	Self-report and expert assessment. SIC		History of work-related asthma symptoms, lung-function test, IgE measurements, SPT
Lastovkova et al (33)	CS	Asthma patients from heat-exchanger production line (N=5)	100%	Other chemical	Workplace measurement of air concentration, SIC		Lung-function test, IgE measurements
Lawrence et al (34)	Cohort	Oil spill response and cleanup workers (N=19 018).	NS	Other chemicals	Full-shift personal air samples		Physician-diagnosed asthma
Le Moual et al (35)	CSS	Asthma patients and population-based subjects (N=683)	41.2%	Other chemicals	Questionnaire, component analysis		Asthma symptoms, lung-function test, IgE measurements, SPT
Lillienberg et al (36)	Cohort	General Nordic population from seven geographic centres (N=13 284)	74%	Different exposures	Self-reported job history combined with an asthma-specific expert-based JEM		Questionnaire: “Do you have or have you ever had asthma after the age of 16” and “Have you ever had asthma diagnosed by a physician”
Lillienberg et al (37)	Cohort	General Nordic population from seven geographic centres (N=13 284)	74%	Different exposures	Self-reported job history combined with two asthma-specific expert-based JEM		Questionnaire: “Do you have or have you ever had asthma after the age of 16” and “Have you ever had asthma diagnosed by a physician”
Lipinska-Ojrzanowska et al (38)	CR	51-year-old process operator working with dishwashing tablets (N=1)	100%	Enzymes	Self-reported exposure to cleaning agents, material data sheets, SIC		History of work-related asthma symptoms, lung function tests, IgE measurements, SPT
Lipinska-Ojrzanowska et al (39)	CS	Cleaners with suspected asthma (N=50)	100%	Other chemicals	Self-reported exposure to cleaning agents, material data sheets, SIC		History of work-related symptoms, lung-function test, IS, IgE measurements, SPT
Liu et al (40)	CSS	Population-based sample of plastic film greenhouse workers (N=5420)	92.2%	Other chemicals	Interview		History respiratory symptoms, lung-function test
Mason et al (41)	CS	Asthma patients in seafood processing sector (N=58)	100%	Different exposures	NS		Chest physician-diagnosed asthma
Mason et al (42)	CR	Spray painter	100%	Other chemicals	SIC		Confirmed asthma based on symptoms, lung function test, FeNO, induced sputum collection and SPT
Migueres et al (43)	CS	Asthma patients (N=111)		Other chemicals	SIC		Confirmed asthma based on symptoms, spirometry, medication
Moore et al (44)	CS	Domestic cleaners and healthcare workers (N=4)	100%	Other chemicals	SIC		Confirmed asthma based on lung-function test, IgE measurements
Oppliger et al (45)	Cohort	Laboratory animal workers/students (N=177)	58,6%	Mammals	Questionnaire (interview), personal workplace measurements (airborne dust)		History of symptoms, lung-function test, IgE measurements
Pacheco Da Silva et al (46)	Cohort	French population-based cohort (N=43 507)	61.0%	Other chemicals	Questionnaire		Questionnaire: Yes to “Have you ever had asthma?” were considered as “ever asthma” and among these, “current asthma” status was classified if yes to asthma symptoms, asthma attacks or asthma treatment were present in the past 12 months.
Patel et al (47)	CSS	Active primary farm operators; responsible for running the farm (N=11 210)	70.8%	Other chemicals	Questionnaire and information on active ingredients obtain through product research page		Physician diagnosed asthma and still symptoms (current asthma)
Patel et al (48)	CSS	Nurse aids at aging and disability services (N=413)	21.6%	Other chemicals	Expert-rated JEM		Questionnaire: “Have you ever had asthma?” and “Has it been confirmed by a doctor?” Asthma and allergy symptoms were based on eight items.
Pravettoni et al (49)	CR	41-year-old food industry worker (N=1)	100%	Mushrooms:	Self-report and expert assessment		History of work-related asthma symptoms, lung-function tests, FeNO, SDS-PAGE, IgE-immunoblotting, IgE measurements, SPT
Roussel et al (50)	CS	Archive workers (N=144)	54%	Moulds fungi or yeast	Measurements; Air and dust samples, questionnaire		Self-reported physician diagnosed asthma
Simoneti et al (51)	CSS	Workers/students working with/without laboratory animals (N=737)	95%	Mammals	Self-report, dust samples from work room floor		History of respiratory symptoms, lung-function tests, SPT
Simoneti et al (52)	CSS	Workers/students working with laboratory animals (N=453)	95%	Mammals	Self-reports		History of respiratory symptoms, lung-function tests, SPT
Singh et al (53)	CSS	Workers from dental institutions (N=454)	NS	Other chemicals	Self-reports		Questionnaire: “Have you had an attack of asthma in the last 12 months” and “Are you currently taking any medication for asthma”, lung-function test, IgE measurement
Sit et al (54)	CC	Population from a French nutritional cohort (N=4469)	54.5%	Other chemicals	Expert-based JEM		Questionnaire: “Have you ever had asthma?” and questions related to symptoms, asthma treatment, and asthma attacks
Song et al (55)	CR	Wallpaper manufacturer (N=1)	100%	Other chemicals	PVC and stone powder handled at workplace, no measurements, SIC		History of respiratory symptoms, lung-function test, IgE measurements, SPT, patch test, sputum eosinophils
Suojalehto et al (56)	CR	Factory workers (N=93)	92%	Other chemicals	Questionnaire, interview, dust measurements, observations, SIC		History of respiratory symptoms, lung-function test, IgE measurements, SPT, open skin application test
Suojalehto et al (57)	CR	Asthma patients with working with epoxy resins/triglycidylether (N=113)	100%	Other chemicals	Workplace measurements, SIC		Symptoms, lung-function test, SPT
Suojalehto et al (58)	CR	Patients with asthma (N=598)	100%	Other chemicals	SIC and placebo control		Lung function test and markers of airway inflammation
Tustin et al (59)	CSS	Workers from manufacturing countertops (N=64)	NS	Other chemicals	Occupational hygienists walk‐throughs, measurements		Lung function test, reversibility testing, medical history, questionnaire
Vandenplas et al (60)	CS	Cleaners (N=44)	100%	Other chemicals	Interview, data sheets, expert assessment, SIC		Lung-function test, sputum cell counts
Vincent et al (61)	CC	Asthma patients with and without mould sensitization (N=64)	NS	Mould, fungi, yeast	Questionnaire, mould contamination assessed and measured in main rooms at home		History of respiratory symptoms, lung-function test, IgE measurements, SPT or cellulose acetate membrane precipitin to moulds
				Definition	Assessment		Assessment
Vizcaya et al (62)	CC	Cleaners (N=95)	49.7%	Other chemicals	Interview		History of respiratory symptoms, lung-function test, IgE measurements
Walters et al (63)	CS	Workers from different industries (N=20)	100%	Other chemicals	Medical/hygiene reporting from companies, SIC		History of respiratory symptoms, lung-function test, IgE measurements, SPT
Walters et al (64)	CS	Clinical patients from different jobs (N=80)	100%	Other chemicals	Medical/hygiene reporting from companies, SIC		History of respiratory symptoms, lung-function test, IgE measurements, SPT
Weinmann et al (65)	Cohort	Population based cohort (N=1695)	22.6%	Other chemicals	Questionnaire		Questionnaire: Physician-diagnosed asthma and either wheezing without cold or use of asthma medication within the last 12 mounts
Weinmann et al (66)	Cohort	Workers in health services or jobs involving cleaning (N=356)	17.3%	Other chemicals	Questionnaire		Questionnaire: Doctor-diagnosed asthma and current asthma
Wittczak et al (67)	CR	Three female nurses (N=3)	100%	Other chemicals	Exposure assessment not specified, SIC		Lung-function test, IgE measurements, SPT sputum

### Relation between potential occupational sensitizing exposures and asthma

Measure of association for each study is presented in supplementary appendix 8. The table also presents the risk of bias assessment and overall confidence in study results for each study, the *a priori* level of evidence based on the overview of systematic reviews (8), and the level of evidence based on the included studies in this review according to the Royal College of General Practitioners' system. The level of evidence for subgroups/specific exposures was only presented if the relation was moderate or strong. As no studies were identified for molluscs, the following section describes the results for the nine studied exposures. Table 2 present an overview of the results found in our overview.

**Table 2:** Overview of the number of new studies and level of evidence for the 10 potential occupational sensitizing exposure groups.

Exposure		Number of new studies	Level of evidence based on overview of systematic reviews (8)	Level of evidence based on the present study
Anhydrides (not Phthalic anhydride)		1	Limited/contradictory	Limited/contradictory
Amines		1	No evidence	Very limited/contradictory*
Biocides		5	No evidence	Very limited/contradictory*
Pesticides		5	Limited/contradictory	Moderate*
Crustaceans (not lobsters, snow crabs)		5	Limited/contradictory	Moderate*
Enzymes**		6	Limited/contradictory	Moderate*
Mammals		8	Very limited/contradictory	Limited/contradictory*
Lab animals		1	Strong	Strong
Metals		11	Very limited/contradictory	Limited/contradictory*
Mould, fungi and yeast		4	Limited/contradictory	Limited/contradictory
Molluscs		0	Limited/contradictory	Limited/contradictory
Other chemicals (not drugs, dyes, biocides, and isocyanates)		33	Limited/contradictory	Limited/contradictory
Cleaning agents		23	Moderate	Moderate
	Bleach	5	Moderate	Moderate
	Chloramine	3	Limited/contradictory	Moderate*
	Disinfection	8	Limited/contradictory	Moderate*
	Formaldehyde	3	Moderate	Moderate
	Glutaraldehyde	6	Moderate	Moderate
	Spray	9	Moderate	Moderate
	Quaternary ammonia compounds	5	Moderate	Moderate
Highly reactive chemicals		5	Limited/contradictory	Limited/contradictory
Unspecified group of other chemicals		12	Limited/contradictory	Moderate*
	Acrylates	3	Limited/contradictory	Moderate*
	Epoxy	4	Limited/contradictory	Moderate*

* The level of evidence has been upgraded. ** Not a-amylase from Aspergillus oryzae, detergent enzymes, Papain, Phytase from Aspergillus niger, various enzymes from Bacillus subtilis (alcalase, protease, maxatase, maxapem, esperase, cellulase, a-amylase, lipase, subtilisin).

### Anhydrides

Anhydrides were studied in 1 low quality-rated case series finding that acid anhydrides induced asthma in 9 persons (43). Based on the inclusion of this additional study, the level of evidence remained unchanged corresponding to limited/contradictory evidence.

### Amines

Amines were studied in 1 low quality-rated case series finding that amines induced asthma in 10 persons (43). Based on the inclusion of one additional study, the level of evidence was upgraded from no evidence to very limited/contradictory evidence.

### Biocides

Pesticides were studied in five low-to-moderate quality-rated studies (20, 24, 31, 40, 47). Statistically significant associations were found for four groups (ie, any pesticide use, herbicides, insecticides, and multiple pesticides) (40, 47), while non-statistically significant associations were found for the remaining pesticides (ie, work with pesticides, glyphosate, paraquat (1,1’-dimethyl-4,4’ bipyridinium dichloride), phenoxy, and 2,4-D) with effect measures between 1.24 (95% CI 1.03–1.49) and 2.18 (95% CI 0.99–4.82) (20, 24, 47). A case series found that pesticides caused asthma in one patient (31). Based on the inclusion of an additional five studies, the level of evidence was upgraded for the main group of biocides to very limited/contradictory, while we upgraded the level of evidence for pesticides from limited/contradictory to moderate.

### Crustaceans

Crustaceans (only shellfish) were studied in five studies with low to high quality-ratings (15, 16, 25, 29, 41). A statistically significant association was found for fish/shellfish in two studies (15, 41), an association was concluded for shellfish powder in a case report (16), an incidence proportion ratio of 12.5 and an incidence ratio of 70 was found in two studies (25, 41), while the final study found no statistically significant association [odds ratio (OR) 1.0 (95% CI 0.4–2.2)] (29). Based on new studies, we upgraded the level of evidence from limited/contradictory to moderate.

### Enzymes

Six studies with quality ratings varying from low to high included enzymes (14, 21, 25, 29, 37, 38). Statistically significant associations were found for two enzymes (ie, antigenic enzymes and enzymes) (21, 25), two case reports found that Bacterial alpha-Amylase termamyl and savinase caused asthma (14, 38), and a non-statistically significant association was found for two enzymes (ie, bioaerosol enzymes and enzymes) with effect measures between 1.3 (95% CI 0.6–3.1) and 2.2 (95% CI 0.3–16.0) (29, 37). Based on the new studies, the level of evidence was upgraded to moderate.

### Mammals

Eight studies with study quality rating varying from low to high were included (four studies of animals) (15, 21, 25, 29, 32, 45, 51, 52). A statistically significant association was found for lab animals (52), one study found that “rat or mouse” exposure caused asthma in six persons (45), a case report concluded that raw pork meat caused asthma in one person (32), while no statistically significant association was found for mouse allergens [risk ratio 1.00 (95% CI 0.5–1.9)] (51). Based on studies of mammals, we upgraded the level of evidence from no scientific to limited/contradictory evidence.

### Metals

Eleven studies with quality rating varying between low and moderate examined the main group of metal (15, 21, 25, 29, 31, 36, 37, 43) and 14 specific metals (13, 30, 55). For the main group of metal, two studies found statistically significant results (ie, “metal and metal fume”, “metal and metal fume antigens”, and “metal working fluids”) (15, 25), one case study found that metals induced asthma in 26 persons respectively (43), a non-statistically significant association was found for “metal and metal fume antigens” [hazard ratio 1.3 (95% CI 0.6–2.6)] (37), while no statistically significant association was found for five groups (ie, metal, metal sensitizers, and metal working fluids) (21, 25, 29, 36). For specific metals, statistically significant associations were found for seven metals (ie, Cd, Cr, Cu, Mo, Ni, Se, U) (30), while one case report found that Nickel (Ni) cased asthma in one person (55). Based on new studies, we upgraded the level of evidence for metals from no evidence to limited/contradictory evidence.

### Mold, fungi and yeast

Mold, fungi and yeast were studied in four low quality-rated studies (17, 49, 50, 61). Oyster mushrooms and shitake mushrooms was found to cause asthma in two case reports (17, 49), non-statistically significant associations were found for “contact with moldy documents” [OR 1.8 (95% CI 0.5–6.8)] and “fungi in arhieves” [OR 1.5 (95% CI 0.5–4.8)] (50), while no statistically significant associations were found for two exposures (ie, *Alternaria alternata* and “fungi in archives”) (50, 61). Based on the new studies, the level of evidence remained limited/contradictory.

### Other chemicals

Other chemicals were studied in 33 low-to-high quality-rated studies, which included over 100 chemicals. We divided other chemicals into three subgroups ie, cleaning agents, highly reactive chemicals, and an unspecified group of other chemicals.

*Cleaning agents:* Cleaning agents were studied in 23 low-to-high quality-rated studies (15, 18, 22, 23, 25, 26, 29) (19, 31, 35–37, 39, 44, 46, 48, 53, 54, 60, 62, 64–66). Associations were found for 69 agents/groups (15, 18, 19, 23, 25, 26, 31, 35–37, 39, 44, 46, 54, 60, 62, 64–66), while no associations were found the remaining agents/groups. Based on new studies, the level of evidence remained moderate for the main group of cleaning agents, but we identified new subgroups with moderate evidence including chloramine and disinfection products.

*Highly reactive chemicals:* Five studies with low quality rating found no statistically significant association for six highly reactive chemicals (21, 25, 29, 36, 37), and therefore did not change the level of evidence (ie, limited/contradictory).

*Unspecified group of other chemicals:* 17 chemicals were studied in 12 studies with low-to-high quality (27, 28, 33, 36, 42, 55–59, 63, 67). An association was found for 15 chemicals [ie, 3-(Bromomethyl)-2-chloro-4-(methylsulfonyl)-benzoic acid, BCMBA, acrylates, alkyl-Cyanoacrylate, “chlorhexidine, epoxy components (ie, epoxy resin, polyamine hardener, triglycidyl iso-Cyanurate), Epoxy resin, sand and phthalic anhydride countertops fabrication”, methyl-Cyanoacrylate, potassium aluminum tetraflouride, Polyfunctional aziridine, polyvinyl Chloride (PVC)]. The level of evidence for this broad group of other chemicals was upgraded to moderate, and we also specifically upgraded acrylates and epoxy to moderate evidence.

## Discussion

### Main results

This systematic review of 55 studies of the relation between asthma and ten potential occupational sensitizing exposure groups was used to update the evidence level found in our recently published overview of systematic reviews (8). No new studies were found for molluscs. For the nine other studied potential occupational sensitizing exposure groups, we upgraded 12 main or subgroups. For main groups, crustaceans and enzymes were upgraded to moderate evidence, mammals and metals to limited/contradictory evidence, and amines and biocides to very limited/contradictory evidence. For subgroups/specific exposures, pesticides, cleaning agents such as chloramine and disinfection products, and an unspecified group of other chemicals such as acrylates and epoxy were upgraded to moderate evidence.

### Methodological considerations

The strengths of our systematic review encompass the comprehensive literature search used to identify all potentially relevant articles and the predefined eligibility criteria established to minimize bias arising from the selective consideration of evidence. Additional strengths include the systematic approach to study exclusion, data extraction, risk of bias assessment, and level of evidence evaluation, conducted independently by two reviewers in a transparent and replicable manner. A potential limitation of the study is the exclusion of gray literature (eg, reports or other non-peer-reviewed literature). We assume that articles of high scientific quality, and therefore the most informative studies, will be published in peer-reviewed journals; consequently, we do not anticipate that the exclusion of gray literature has significantly influenced our conclusion. However, the possibility of publication bias cannot be excluded. This bias may result in an overrepresentation of positive or significant findings, potentially leading to an inaccurate assessment of the level of evidence. A further limitation is the substantial heterogeneity among studies, which precluded meta-analysis. The heterogeneity is especially found in the definition of asthma. The use of different diagnostic criteria reduces comparability between studies and may lead to outcome misclassification, especially if the criteria rely solely on symptoms. Consequently, the lack of standardized asthma classification in the included studies may to some extents have influenced our conclusion. Finally, several of the studies included few participants, reducing the statistical power, making it more difficult to detect potential relations and limiting the generalizability of the results.

Due to resource constraints, this study did not encompass all potential occupational sensitizing exposures with limited or no evidence. Specific criteria were employed for the inclusion of ten potential occupational sensitizing exposure groups, namely frequent exposures, suspected low-molecular-weight sensitizing exposures (six out of ten included exposure groups), and sensitizing exposures not widely recognized as established causes of asthma. Frequently used exposures were prioritized due to the potentially greater impact of preventive measures. Low molecular weight exposures were emphasized due to the less-well understood mechanisms and the lack of diagnostic tools associated with these agents (68). Additionally, we prioritized exposures currently subject to debate among clinicians and researchers, such as pesticides and epoxy resins.

The objective of the study was to evaluate the evidence for potential occupational sensitizing exposures and asthma; consequently, studies of exposures known to be irritants without any indication of a specific immunological mechanism, such as ammonia, were excluded. However, main groups and subgroups/specific exposures comprising a mixture of irritants and sensitizing exposures were included to ensure comprehensiveness. As a result, irritants without a specific mechanism of action have to some extend been incorporated, for example for cleaning agents. Furthermore, certain main groups (eg, metals) encompassed heterogeneous exposures (eg, metal working fluids), but these heterogeneous exposures were included to maintain a comprehensive approach.

Case studies are in general considered as low evidence for causal inference. However, we believe that well-performed asthma case reports with a specific inhalation challenge (SIC) can be regarded as an experiment without confounding issues and with accurate exposure and outcome data. Therefore, case reports can provide detailed, high-quality data that can help identify occupational sensitizing exposures and enhance the understanding of the mechanisms underlying asthma development. In addition, including case reports in our systematic review allows for the identification of rare or novel associations between occupational sensitizing exposures and asthma that may not be captured in larger observational studies. Consequently, we developed a novel risk of bias assessment tool for a joint evaluation of observational and case studies. A priori, the tool does not confer preference to any of the study types. We anticipate that this tool will prove valuable in future studies on risk factors for asthma.

Using our risk of bias assessment tool, studies relying on self-reported information of exposure and/or outcome were classified as having a potential risk of bias. Self-reports were typically found in observational studies, where differential misclassification especially may occur in cross-sectional and case–control studies. Consequently, these studies were generally assigned a low confidence in study results. The use of blinded specific inhalation challenges (SIC) was assessed as having low risk of bias, although the potential for false-positive and false-negative results remains a risk.

### Discussion of results

The confidence of study results of the included studies varied from low to high; however, it was still possible to upgrade the evidence level for additional potential occupational sensitizing exposures, highlighting the advantages of a systematic approach. New main groups with moderate evidence include crustaceans and enzymes, while new subgroups/specific exposures with moderate evidence encompass pesticides, chemicals/cleaning agents such as chloramine and disinfection products, and an unspecified group of other chemicals, including acrylates and epoxy resins. Based on our overview and systematic review, we found strong evidence of a causal relation for the main groups of wood dust and moderate evidence for the main groups of mites, fish, crustaceans, and enzymes. For subgroups/specific exposures, we found strong evidence of exposure to various laboratory animals and moderate evidence for 60 subgroups/specific exposures.

The study incorporated both the main exposure groups and subgroups/specific exposures. The subgroups/specific exposures were primarily examined in case studies utilizing SIC tests, while the main groups of exposures were investigated in observational studies, often using self-reports or job exposure matrices. Case studies have provided substantial to the evidence level for several subgroups/specific exposures. These studies contribute to the understanding of the impact of specific exposures (eg, alpha-amylase) on asthma and serve as a robust tool in the causal evaluation of an asthma patient. The knowledge derived from the main groups of exposures provides a broader comprehension of the population prevalence, risk, and exposure–response relation between groups of exposure (eg, wood dust) and asthma, which is relevant for both clinical and preventive evaluations. For preventive strategies, knowledge of the main groups facilitates generalized advice for prevention and avoidance, such as recommending methods to reduce wood dust exposure without specifying specific types of wood. However, we did not find solid evidence of a causal relation between several well-known main groups of exposures. For example, it is generally accepted that numerous animal proteins can cause IgE-mediated allergies and eventually cause asthma. In this review, we found limited/contradictory evidence of a relation for mammals. The lack of evidence for the main group of mammals may, among others, be explained by not including cows and rats in this review [due to high evidence in our overview of systematic review (8)]. Furthermore, sensitizing exposures that are known to cause asthma, for example, from cats and dogs, are seldom investigated in occupational studies.

The ambition of this review was in a transparent and systematic way to update the current evidence level for potential occupational sensitizing exposure groups using the results from a recent overview (8). This resulted in an efficient and comprehensive evaluation of the existing literature. Baur's et al (7) extensive systematic review was influential on the results in the overview. Of note, in our systematic review, we did not use the same tool for evaluating the risk of bias as Baur; most importantly, we assigned more weight to high-quality case studies. Therefore, we might have found moderate-to-strong evidence for even more potential occupational sensitizing exposures if we had used our risk of bias tool on the several hundred studies included in Baur et al's review (7).

### Perspectives

Our systematic review offers a comprehensive overview of the existing literature including the level of evidence of the relation between ten potential occupational sensitizing exposure groups and asthma. Including ten exposures in one article provides a broader understanding of suspected occupational causes to asthma and facilitates comparisons of evidence level across multiple exposures in a single resource. The inclusion of both observational and case studies, each possessing distinct epidemiological strengths, enhances the understanding of occupational asthma, while simultaneously highlighting the limitations within the existing literature, particularly regarding the scarcity of research on well-established sensitizing exposure groups.

The identification of occupational sensitizing exposures able to cause asthma is of critical importance, particularly in the context of accurate diagnosis, targeted prevention and highlighting existing knowledge gaps. The identification is essential as it not only establishes a clear link between these exposures and the development of asthma, but also provides valuable insights into the underlying mechanisms of the disease. Therefore, our systematic review can be a valuable tool for causal evaluation of asthma patients. Also, the review will support a proper risk assessment for compagnies and industries, where potential sensitising exposures might be present. Furthermore, the review will target preventive measures in the workplace in relation to exposure reduction and regulatory actions, which are crucial for preventing the development or progression of asthma among workers. Finally, our review highlights knowledge gaps in the existing literature, which underscore the need for further studies to strengthen the identification of potential sensitising exposures.

### Concluding remarks

In this systematic review, we included 55 studies from ten groups of occupational exposures. We were able to update the evidence level for twelve main groups, subgroups, and specific occupational sensitizing exposures as related to asthma. New main groups with moderate evidence includes crustaceans and enzymes, while new subgroups/specific exposures with moderate evidence includes pesticides, chemicals/cleaning agents such as chloramine and disinfection products, and unspecified group of other chemicals such as acrylates and epoxy. Our systematic review can be a valuable tool to ensure accurate asthma diagnosis, facilitate targeted prevention at the workplace level, and highlighting knowledge gaps in the existing literature.

## Supplementary material

Supplementary material
