# Supplementary material for: A systematic review of the relation between ten potential occupational sensitizing exposures and asthma
Source: Scand J Work Environ Health. 2025 Apr 27;51(3):146–58. doi: 10.5271/sjweh.4214 (PMC12047181; doi:10.5271/sjweh.4214)
Supplement: Supplementary material [file SJWEH-51-146-S001.pdf]

A systematic review of the relation between ten potential occupational sensitizing exposures and asthma[1]

by Annett Dalbøge, PhD, Henrik Albert Kolstad, PhD, Alexander Jahn, MSc, Charlotte Suppli Ulrik, PhD, David Lee Sherson, Harald William Meyer, PhD, Niels Ebbehøj, PhD, Torben Sigsgaard, PhD, Xaver Baur, PhD, Vivi Schlünssen, PhD

1. Supplementary material
2. Correspondence to: Annett Dalbøge, MSc, PhD, Department of Occupational Medicine, Danish Ramazzini Center, Aarhus University Hospital, Palle Juul-Jensens Boulevard 99, 8200 Aarhus N, Denmark. [E-mail: annett.dalboege@rm.dk]

Appendix 1: PECO for peer-reviewed studies 1
Appendix 2: Full search string for literature search in PudMed/MEDLINE 3
Appendix 3: Study exclusion criteria 4
Appendix 4: Risk of bias assessment 5
Appendix 5. Modification of the Royal College of General Practioners three star system 7
Appendix 6: List of 98 excluded studies and the reason for exclusion 8
Appendix 7. Study characteristics and risk of bias of the 55 included studies 21
Appendix 8. Measure of association and conclusion of the 55 included studies 32

Appendix 1: PECOS

Population
Adult human population (in or above working age)

Exposure
Exposure definition: Primary exposures include occupational exposures suspected to cause asthma (hereafter called allergens). The allergens include both high- and certain low-molecular-weight agents, which will be divided into the following groups;

- Amines
- Anhydrides (not Phthalic anhydride)
- Biocides
- Crustaceans (not lobsters and snow crabs)
- Enzymes (not a-amylase from *Aspergillus oryzae*, detergent enzymes, Papain, Phytase from *Aspergillus niger*, various enzymes from *Bacillus subtilis* (alcalase, protease, maxatase, maxapem, esperase, cellulase, a-amylase, lipase, subtilisin)
- Mammals (not cows, rats)
- Metals (not Platinum salts)
- Mold, fungi and yeast (not Aspergillus, Cladosporium, Penicllium)
- Molluscs
- Other chemicals (not drugs, dyes, biocides, and isocyanates)

Exposure assessment: We include studies with exposure information based on subjective (self-reports) or objective (e.g., expert based, observations, technical measurements) assessment, and where exposure estimates are quantified ranging from dichotomous to continuous variables. Studies with more proxy measure of exposures such as job title or industry (no exposure quantification) will not be included.

Comparison
We include studies with exposure contrast between persons/groups (e.g., exposed vs. non/low exposed). Only studies, where a measure of association of the effect of allergens on asthma has been estimated or is possible to estimate will be included.

Outcome
Outcome definition: Outcome will be defined as asthma. Asthma is considered a common chronic disorder of the airways that is complex and characterized by variable and recurring symptoms, airflow obstruction, bronchial hyper-responsiveness, and an underlying inflammation. Outcome will not include pre-existing asthma aggravated by work (might be difficult to evaluate).

Outcome assessment: In observational studies, we will include studies, where outcome is assessed as:

- Self-reported by workers, or
- Clinical diagnosis as reported by workers, or
- Clinical diagnosis by medical expert, or

Objective measurements: spirometry (LFT, sPET), immunology (as marker of effect), and provocation (lung) as the latter verification by experimental condition will be more specific

In case-reports, we will only include studies where outcome assessment is based on self-reported symptoms in combination with objective measurements (e.g., spirometry (LFT, sPET), immunology (as marker of effect), or provocation (skin, lung)).

Appendix 2: Full search string for literature search in PudMed/MEDLINE

(((((((((((((((((((((((((((((((signs and symptoms, respiratory[MeSH Terms]))) OR ((allergy and immunology[MeSH Terms]))) OR allergy[Text Word]) OR immunology[Text Word]) OR "respiratory function tests"[MeSH Terms]) OR respiratory function test*[Text Word]) OR pulmonary function test*[Text Word]) OR lung function test*[Text Word]) OR bronchial hyperreactivity[MeSH Terms]) OR bronchial hyperreactivit*[Text Word]) OR airway obstruction[MeSH Terms]) OR airway obstruction[Text Word]) OR respiratory hypersensitivity[MeSH Terms]) OR airway hyper responsiveness[Text Word]) OR respiratory hyper responsiveness[Text Word]) OR respiratory hypersensitivit*[Text Word]) OR lung diseases, obstructive[MeSH Terms]) OR obstructive lung disease*[Text Word]) OR obstructive pulmonary disease*[Text Word]) OR asthma[MeSH Terms]) OR asthma, occupational[MeSH Terms]) OR occupational asthma*[Text Word])) **AND** (((((((((((((workplace[MeSH Terms]) OR occupational exposure[MeSH Terms]) OR occupational diseases[MeSH Terms]) OR occupational exposure*[Text Word]) OR work related[Text Word]) OR workplace*[Text Word]) OR work environment[Text Word]) OR job site*[Text Word]) OR work location*[Text Word]) OR work site*[Text Word]) OR work place*[Text Word]) OR occupation[Text Word]) OR work[MeSH Terms])) **AND** (((((((((((((((((((allergens[MeSH Terms]) OR allergic[Text Word]) OR allergen*[Text Word]) OR antigen*[Text Word]) OR antigens[MeSH Terms]) OR hypersensitivit*[Text Word]) OR immediate[Text Word]) OR ige[Text Word]) OR immunoglobulin e[Text Word]) OR sensation[MeSH Terms]) OR hypersensitivity, immediate[MeSH Terms]) OR ((bites and stings[MeSH Terms]))) OR fungi[MeSH Terms]) OR dust[MeSH Terms]) OR feathers[MeSH Terms]) OR sensitization[Text Word]) OR sensitisation[Text Word]) OR asthmogen*[Text Word]) OR asthmagen*[Text Word])) **AND** ((((((((((((((((((causality[MeSH Terms]) OR etiology[MeSH Terms]) OR risk factors[MeSH Terms]) OR etiology[MeSH Subheading]) OR causation[Text Word]) OR causing[Text Word]) OR causalit*[Text Word]) OR etiolog*[Text Word]) OR epidemiolog*[Text Word]) OR reinforcing factor*[Text Word]) OR enabling factor*[Text Word]) OR predisposing factor*[Text Word]) OR odds ratio[MeSH Terms]) OR odds ratio*[Text Word]) OR risk[MeSH Terms]) OR incidence proportion rate*[Text Word]) OR incidence[MeSH Terms]) OR incidence rate*[Text Word]))) NOT ((animals[MeSH Terms]) NOT humans[MeSH Terms]))) **AND** ((danish[Language]) OR english[Language])

Appendix 3: Study exclusion criteria

1) The following exclusion criteria were used at title/abstract screening:

- No indication of asthma or "unspecific lung diseases" as outcome measure, or
- No indication of occupational allergens as exposure, or
- No indication of evaluation of association between asthma and occupational allergens, or
- Other reasons:
  - Animal study, children/student study, or
  - A systematic review; abstract should indicate that the literature search was performed systematic
  - Not English/Danish language
  - Books, letters to editor, reviews, abstracts only will be excluded

2) The following exclusion criteria were used at full paper reading:

- Study outcome does not fulfill outcome definition (i.e., outcome definition in PECO), or
- Study exposures does not fulfill exposure definition (i.e., exposure definition in PECO), or
- No indication of evaluation of association between asthma and occupational allergens, or
- Other reasons
  - Animal study, children/student study, or
  - A systematic review; no indication of a systematic literature search
  - Not English/Danish language
  - Books, letters to editor, reviews, abstracts only will be excluded

Appendix 4: Risk of bias assessment

Item		Objective	Scoring	Comment
**Design and study population**				
1	Study design	Basic principle for causal inference	• Experimental: 1<br>• Observational: 0	• Experimental studies with specific inhalation challenge (SIC) of individual participants or intervention studies=1<br>• Other epidemiological studies=0
2	Population	Asses risk factors of incident asthma	• Population at risk: 1<br>• Patients: 0	• Epidemiological studies of incident asthma=1<br>• Other types of studies: SIC in case reports will by definition only include prevalent asthma=0
3	Participation rate	Assess potential for selection bias	• High: 1<br>• Low: 0	• ≥60%=1<br>• <60%, unknown or case-reports=0
**Exposure**				
4	Exposure specificity	Assess specific exposure	• High: 1<br>• Low: 0	• Specific allergens, chemicals, plants, animals=1<br>• Task, product, process, occupation, industry=0
5	Exposure assessment - I	Assess potential for information bias	• Blinded/objective: 1<br>• Not blinded/ subjective: 0	• Blinded SIC with relevant sham=1<br>• Blinded SIC with no sham=0<br>• Observational study with independent exposure assessment=1<br>• Self-reported exposure=0
6	Exposure assessment - II	Individual or group-based assessment	• Individual assessment:1<br>• Group-based assessment: 0	• Self-reports, measurements on the individual level, employment records=1<br>• JEMs or other group-based approaches=0
**Outcome**				
7	Outcome assessment	Assess potential for information bias	• Clinical: 1<br>• Other: 0	• Objective measurements of variability of lung function (e.g. reversibility, bronchial hyperreactivity or peak flow)=1<br>• Other=0

**Association**				
8	Confounders adjusted for	Assess potential for confounding	• Appropriate: 1<br>• Inappropriate: 0	• Observational studies: smoking, age, BMI and atopy (adjustment or stratification), case-reports with SIC if sham=1 (own control)<br>• Other=0
9	Exposure-response relation	Core criterion for causality	• Assessed: 1<br>• Not assessed: 0	• At least 3 levels of exposure or continuous exposure metric=1<br>• Other=0

Item 10: Rating overall confidence in the results of the study

HIGH - Zero or one non-critical weakness:
The study provides an accurate estimate of the association between exposure and outcome.

MODRATE - More than one non-critical weakness*:
The study has more than one weakness, but no critical flaws. It may provide an accurate estimate of the association between exposure and outcome.

LOW - One critical flaw with or without non-critical weaknesses:
The study has a critical flaw and may not provide an accurate estimate of the association between exposure and outcome

CRITICALLY LOW - More than one critical flaw with or without non-critical weaknesses:
The study has more than one critical flaw and should not be relied on to provide an accurate estimate of the association between exposure and outcome

***Note:** *Multiple non-critical weaknesses may diminish confidence in the study and it may be appropriate to move the overall appraisal down from moderate to low confidence

Risk of bias assessment including 10 items. Item 1-9 was scored "High" or "Low", while item 10 could be scored "High", "Moderate", Low" or "Critically low".

Appendix 5. Modification of the Royal College of General Practioners three star system (RCGP)[6]

Strong evidence	- Provided by generally consistent findings in multiple, high-quality scientific studies<br><br>Note: Consistent findings in moderate to high quality-rated studies are required. If moderate to high quality-rated studies exist, low quality-rated case studies with no association can be disregarded.
Moderate evidence	- Provided by generally consistent findings in fewer, smaller or lower quality scientific studies, or<br>- Provided by generally consistent findings in fewer, smaller or lower quality scientific studies, based on questionnaire conducted studies or other weak evidence (clinical weakness (absence of LFT, sPFT, SIC).<br><br>Note: Consistent findings in low to moderate quality-rated studies are required. If several low to moderate quality-rated studies exist, few low case quality-rated studies with no association can be disregarded.
Limited or contradictory evidence	- Provided by one scientific study (analytic) or inconsistent findings in multiple scientific studies, or<br>- Provided by one scientific study based on questionnaires or other weak evidence (clinical weakness (absence of LFT, sPFT, SIC))
Very limited or contradictory evidence	- Provided by at least three case reports, one case series, one non-analytic study or one occupational disease statistic study with at least five asthma cases
No scientific evidence	- No scientific evidence—based on clinical studies, theoretical considerations and/or clinical consensus

When evaluating "*consistent findings*", we disregarded single case studies with no association. A single case study with no association doesn't reject a potential causal association.

Appendix 6: List of 98 excluded studies and the reason for exclusion

Excluded studies		Reason for exclusion
1	Ahmad, I; Balkhyour, MA. Occupational exposure and respiratory health of workers at small scale industries. Saudi Journal Of Biological Sciences 2020;27(3):985-990.	Wrong outcome
2	Ahmed, Amani Shawki; Ibrahim, Dalia Anas; Hassan, Tarek Hamdy; Abd-El-Azem, Wael Galal. Prevalence and predictors of occupational asthma among workers indetergent and cleaning products industry and its impact on quality of lifein El Asher Men Ramadan, Egypt. Environ Sci Pollut Res Int 2022;29(23):33901-33908.	Wrong exposure
3	Anderson, Naomi J.; Reeb-Whitaker, Carolyn K.; Bonauto, David K.; Rauser, Edmund. Work-Related Asthma in Washington State Journal of Asthma 2011;48(8):773-782.	Wrong outcome
4	Ayaaba E.; Li Y.; Yuan J.; Ni C. Occupational respiratory diseases of miners from two gold mines in Ghana. International Journal of Environmental Research and Public Health / 2017;14(3):337.	Wrong outcome
5	Balbay, Ege Gulec; Toru, Umran; Arbak, Peri; Balbay, Oner; Suner, Kezban Ozmen; Annakkaya, Ali Nihat. Respiratory symptoms and pulmonary function tests in security and safety products plant workers. International Journal of Clinical and Experimental Medicine 2014;7(7):1883-1886.	Wrong outcome
6	Baldi, Isabelle; Robert, Celine; Piantoni, Florence; Tual, Severine; Bouvier, Ghislaine; Lebailly, Pierre; Raherison, Chantal. Agricultural exposure and asthma risk in the AGRICAN French cohort. International journal of hygiene and environmental health 2014;217(4-5):435-442.	Wrong exposure
7	Bauer, Andrea; Pesonen, Maria; Brans, Richard; Caroppo, Francesca; Dickel, Heinrich; Dugonik, Aleksandra; Larese Filon, Francesca; Geier, Johannes; Gimenez-Arnau, Ana M; Napolitano, Maddalena; Patruno, Cataldo; Rustemeyer, Thomas; Simon, Dagmar; Schuttelaar, Marie L A; Spiewak, Radoslaw; Stingeni, Luca; Vok, Marko; Weisshaar, Elke; Wilkinson, Mark; Valiukeviciene, Skaidra; Uter, Wolfgang. Occupational contact allergy: The European perspective-Analysis of patchtest data from ESSCA between 2011 and 2020. Contact Dermatitis 2023; not further specified.	Wrong outcome
8	Beijer, E; Meek, B; Bossuyt, X; Peters, S; Vermeulen, R C H; Kromhout, H; Veltkamp, M. Immunoreactivity to metal and silica associates with sarcoidosis in Dutchpatients. Respir Res 2020;21(1):141.	Wrong outcome
9	Betancor, Diana; López-Matas, María A; González-Ruiz, Azahara; Martín-López, Laura; Carnés, Jerónimo; Fernández-Nieto, Maria Del Mar. Sarcoplasmic calcium-binding protein and alcohol dehydrogenase, newoccupational allergens in the fruit fly Drosophila melanogaster. Clin Exp Allergy 2021;51(10):1387-1390.	Wrong outcome
10	Blanc P.D.; Annesi-Maesano I.; Balmes J.R.; Cummings K.J.; Fishwick D.; Miedinger D.; Murgia N.; Naidoo R.N.; Reynolds C.J.; Sigsgaard T.; Toren K.; Redlich C.A. The occupational burden of nonmalignant respiratory diseases an official American thoracic society and european	Other reasons

	respiratory society statement. American Journal of Respiratory and Critical Care Medicine / 2019;199(11):1312-1334.	
11	Cauz, Paola; Bovenzi, M.; Filon, Francesca Larese. Laboratory animal allergy: follow-up in a research centre. Medicina Del Lavoro 2014;105(1):30-36.	Other reasons
12	Chatti, S.; Maoua, M.; Rhif, H.; Dahmoul, M.; Abbassi, A.; Mlaouah, A. J.; Salah, H. Hadj; Debbabi, F.; Mrizak, N. Occupational asthma in the Tunisian central region: Etiologies and professional status. Revue de pneumologie clinique 2011;67(5):281-288.	Other reasons
13	De Olim C.; Begin D.; Boulet L.-P.; Cartier A.; Gerin M.; Lemiere C. Investigation of occupational asthma: Do clinicians fail to identify relevant occupational exposures? Canadian Respiratory Journal / 2015;22(6):341-347.	No association
14	De Troeyer K.; De Man J.; Vandebroek E.; Vanoirbeek J.A.; Hoet P.H.; Nemery B.; Vanroelen C.; Casas L.; Ronsmans S. Identifying cleaning products associated with short-term work-related respiratory symptoms: A workforce-based study in domestic cleaners. Environment International / 2022;162.	Wrong outcome
15	Dumas, Orianne; Le Moual, Nicole; Siroux, Valerie; Heederik, Dick; Garcia-Aymerich, Judith; Varraso, Raphaelle; Kauffmann, Francine; Basagana, Xavier. Work related asthma. A causal analysis controlling the healthy worker effect. Occupational and environmental medicine 2013;70(9):603-610.	Wrong outcome
16	Dumas O.; Varraso R.; Boggs K.M.; Quinot C.; Zock J.-P.; Henneberger P.K.; Speizer F.E.; Le Moual N.; Camargo C.A. Association of Occupational Exposure to Disinfectants with Incidence of Chronic Obstructive Pulmonary Disease among US Female Nurses. JAMA Network Open / 2019 (not further specified).	Wrong outcome
17	Dumas, Orianne; Bédard, Annabelle; Marbac, Matthieu; Sedki, Mohammed; Temam, Sofia; Chanoine, Sébastien; Severi, Gianluca; Boutron-Ruault, Marie-Christine; Garcia-Aymerich, Judith; Siroux, Valérie; Varraso, Raphaëlle; Le Moual, Nicole. Household Cleaning and Poor Asthma Control Among Elderly Women. J Allergy Clin Immunol Pract 2021;9(6):2358-2365.e4.	No association
18	Ecin S.M.; Sandal A.; Cetintepe S.P.; Koyuncu A.; Kurt O.K.; Yildiz A.N.; Demir A.U. Prevalence and Risk Factors of Work-Related Asthma in Hospital Cleaning Workers. Turkish Thoracic Journal / 2022;23(3):203-209.	Wrong exposure
19	Feary, Johanna; Fitzgerald, Bernadette; Schofield, Susie; Jones, Meinir; Cullinan, Paul. Sensitisation to mouse allergens in contemporary laboratory animal workers: The SPIRAL study. European Respiratory Journal 2016;48(Journal Article).	Other reasons
20	Ferraz E.; Simoneti C.S.; Rodrigues M.; Freitas A.S.; Arruda L.K.; Bagatin E.; Vianna E.O. Association of dust allergen in animal laboratories and atopic sensitization . American Journal of Respiratory and Critical Care Medicine 2015;191(Meeting Abstracts).	Other reasons
21	Francuz, B.; Demange, V.; Mousel, M. -L; Grzebyk, M.; Nicaise, P.; Chollet-Martin, S.; Choudat, D. Allergic or irritative symptoms in	Other reasons

	preparation laboratory and animal facilities personnel in a research institute. Archives Des Maladies Professionnelles Et De L Environnement 2014;75(2):126-134.	
22	Galli, Luigina; Facchetti, Susanna; Raffetti, Elena; Donato, Francesco; D'Anna, Mauro. Respiratory diseases and allergic sensitization in swine breeders: a population-based cross-sectional study. Annals of Allergy Asthma & Immunology 2015;115(5):402-407.	Wrong outcome
23	Garrido A.N.; House R.; Lipszyc J.C.; Liss G.M.; Holness D.L.; Tarlo S.M. Cleaning Agent Usage in Healthcare Professionals and Relationship to: Lung and Skin Symptoms. The Journal of asthma: official journal of the Association for the Care of Asthma / 2021.	Wrong exposure
24	Graff, Pal; Bryngelsson, Ing-Liss; Fredrikson, Mats; Flodin, Ulf. Adult onset asthma in non-allergic women working in dampness damaged buildings: A retrospective cohort study. American Journal of Industrial Medicine 2019;62(4):357-363.	Wrong exposure
25	Guarnieri G.; Cattoni I.; Barbetta G.; Liviero F.; Mason P.; Scarpa M.C.; Maestrelli P. Features of occupational asthma in Northern Italy from 1987 to 2012. European Respiratory Journal / 2013;42(SUPPL. 57).	Other reasons
26	Harris-Roberts J.; Robinson E.; Fishwick D.; Fourie A.; Rees D.; Spies A.; Curran A.; Sen D.; Barber C. Sensitization and symptoms associated with soybean exposure in processing plants in South Africa. American Journal of Industrial Medicine / 2012;55(5):458-464.	Wrong exposure
27	Hawley, Brie; Cummings, Kristin J.; Mohammed, Mohammed; Dimmock, Anne E.; Bascom, Rebecca. Allergic sinusitis and severe asthma caused by occupational exposure to locust bean gum: Case report. American Journal of Industrial Medicine 2017;60(7):658-663.	Wrong exposure
28	Henneberger P.; Liang X.; Lillienberg L.; Dahlman-Hoglund A.; Toren K.; Andersson E. Association of asthma exacerbation with objective and subjective assessments of occupational exposure. European Respiratory Journal / 2014;44(SUPPL. 58).	Other reasons
29	Hiller J.; Greiner A.; Drexler H. Respiratory afflictions during hairdressing jobs: case history and clinical evaluation of a large symptomatic case series. Journal of Occupational Medicine and Toxicology / 2022;17(1):10.	Wrong outcome
30	Jaiyesimi A.; Agbaje S. Respiratory symptoms and lung function indices of poultry workers and age-matched apparently healthy individuals in Ibadan, Nigeria. Physiotherapy (United Kingdom) / 2015;101(SUPPL. 1):eS667-eS668.	Other reasons
31	Jones, M.; Welch, J.; Turvey, J.; Cannon, J.; Clark, P.; Szram, J.; Cullinan, P. Prevalence of sensitization to 'improver' enzymes in UK supermarket bakers. Allergy 2016;71(7):997-1000.	Wrong outcome
32	Kammoolkon, Ratanee; Taneepanichskul, Nutta; Taneepanichskul, Surasuk. Respiratory symptoms and their association with exposure to respiratorydust among indigo-dyed cotton workers. Arch Environ Occup Health 2021;77(5):356-361.	Wrong exposure

33	Kim D.; Kim B.; Lee K.; Shin J. A case of occupational asthma occurred in the subway maintenance worker. European Respiratory Journal / 2015;46(SUPPL. 59).	Other reasons
34	Kwizera R.; Bongomin F.; Olum R.; Meya D.B.; Worodria W.; Bwanga F.; Fowler S.J.; Gore R.; Denning D.W.; Kirenga B.J. Fungal asthma among Ugandan adult asthmatics. Medical mycology / 2021; Not further specified.	Wrong exposure
35	Kwon S.-C.; Song J.; Kim Y.-K.; Calvert G.M. Work-related asthma in Korea - findings from the Korea Work-Related Asthma Surveillance (KOWAS) program, 2004-2009. Allergy, Asthma and Immunology Research / 2014;7(1):51-59.	No association
36	Laborde-Casterot, Herve; Rosenberg, Nicole; Dupont, Patricia; Garnier, Robert. Is the Incidence of Aliphatic Amine-Induced Occupational Rhinitis and Asthma Underestimated? American Journal of Industrial Medicine 2014;57(12):1303-1310.	Wrong outcome
37	Laborde-Casterot, Herve; Villa, Antoine F.; Rosenberg, Nicole; Dupont, Patricia; Lee, Hwee Min; Garnier, Robert. Occupational rhinitis and asthma due to EDTA-containing detergents or disinfectants. American Journal of Industrial Medicine 2012;55(8):677-682.	Wrong outcome
38	Laditka J.N.; Laditka S.B.; Arif A.A.; Hoyle J.N. Work-related asthma in the USA: nationally representative estimates with extended follow-up. Occupational and environmental medicine / 2020 (not further specified).	Wrong exposure
39	Laszlo, Endre. Occupational asthma in Hungary. Orvosi hetilap 2015;156(19):769-778.	Other reasons
40	Le Moual N.; Bedard; Dumas; Varraso; Kauffmann; Zock. Relevance of exposure to cleaning agents beyond cleaning professionals: Private homes and healthcare workers. Occupational and Environmental Medicine / 2013;70(SUPPL. 1).	Other reasons
41	Liccardi, Gennaro; Emenius, Gunnel; Merritt, Anne-Sophie; Salzillo, Antonello; D'Amato, Maria; D'Amato, Gennaro. Direct and Indirect Exposure to Horse: Risk for Sensitization and Asthma. Current Allergy and Asthma Reports 2012;12(5):429-437.	Wrong outcome
42	Lindström, Irmeli; Ryhänen, Anna-Mari; Jungewelter, Soile; Suojalehto, Hille; Suuronen, Katri. Asthma onset after exposure to fluorinated hydrocarbons in the presence of combustion. Am J Ind Med 2020;63(11):1054-1058.	Wrong exposure
43	Lipinska-Ojrzanowska A.A.; Wiszniewska M.; Nowakowska-Swirta E.; Walusiak-Skorupa J.M. Airways inflammation in work-related asthma due to high and low molecular weight agents. Allergy: European Journal of Allergy and Clinical Immunology / 2017;72(Supplement 103):240-241.	Other reasons
44	Lipinska-Ojrzanowska A.; Wiszniewska M.; Walusiak-Skorupa J. Work-related asthma in cleaners. Allergy: European Journal of Allergy and Clinical Immunology / 2014;69(SUPPL. 99):116.	Other reasons
45	Lipinska-Ojrzanowska, Agnieszka; Wiszniewska, Marta; Swierczynska-Machura, Dominika; Wittczak, Tomasz; Nowakowska-Swirta, Ewa; Palczynski, Cezary; Walusiak-Skorupa, Jolanta. Work-	Wrong outcome

	related respiratory symptoms among health centres cleaners: A cross-sectional study. International journal of occupational medicine and environmental health 2014;27(3):460-466.	
46	Lucas D.; Robin C.; Vongmany N.; Dewitte J.D.; Lodde B.; Pougnet R.; Larabi L. Main Causal Agents of Occupational Asthma in France, Reported to the National Network for Occupational Disease Vigilance and Prevention (RNV3P) 2001-2018. Annals of work exposures and health / 2022.	No association
47	Malo, J. -L; Ghezzo, H.; L'Archeveque, J. Distinct temporal patterns of immediate asthmatic reactions due to high- and low-molecular-weight agents. Clinical and Experimental Allergy 2012;42(7):1021-1027.	Wrong outcome
48	Marchetti N.; Garshick E.; Kinney G.L.; McKenzie A.; Stinson D.; Lutz S.M.; Criner G.J. Risk of moderate to severe COPD and chronic respiratory symptoms attributable to occupational exposure is similar for men and women in COPDgene. American Journal of Respiratory and Critical Care Medicine / 2013;187(MeetingAbstracts).	Other reasons
49	Mazurek J.M.; White G.E.; Rodman C.; Schleiff P.L. Farm work-related asthma among US primary farm operators. Journal of agromedicine / 2015;20(1):31-42.	Wrong exposure
50	Meza, Francisco; Chen, Lilia; Hudson, Naomi. Investigation of Respiratory and Dermal Symptoms Associated With Metal Working Fluids at an Aircraft Engine Manufacturing Facility. American Journal of Industrial Medicine 2013;56(12):1394-1401.	Wrong outcome
51	Mezni, A. Benzarti; Babay, S.; Ben Jemaa, A. Occupational asthma to the pyrolysis products of plastics in a company manufacturing protective respiratory masks. About 2 cases. Revue Francaise D Allergologie 2012;52(7):474-479.	Other reasons
52	Mezni, A. Benzarti; Guissi, R.; Hsinet, J.; Ben Maiz, N.; Essid, D.; Hamdouni, M.; Ben Jemaa, A. Analysis of acknowledged occupational asthma records over a period of 15 years in a population in northern Tunisia. Revue Francaise D Allergologie 2018;58(6):427-436.	Other reasons
53	Minov J.; Karadzinska-Bislimovska J.; Stoleski S.; Mijakoski D.; Atanasovska A. Distribution of sensitizer-induced occupational asthma in R. Macedonia by occupation in the period 2005-2017. European Respiratory Journal / 2018;52(Supplement 62).	Other reasons
54	Moghtaderi, M.; Farjadian, S.; Hasiri, M. Abbaszadeh. Animal allergen sensitization in veterinarians and laboratory animal workers. Occupational Medicine-Oxford 2014;64(7):516-520.	Wrong outcome
55	Moghtaderi, Mozhgan; Farjadian, Shirin; Hosseini, Zeynab; Raayat, Alireza. Increased Risk of Horse Sensitization in Southwestern Iranian Horse Riders. International journal of occupational medicine and environmental health 2015;28(5):909-913.	Wrong outcome
56	Money A.; Carder M.; Hayes J.P.; Noone P.; Bourke P.; Hayes J.; Agius R. Work related respiratory ill health (WRRIH): Republic of Ireland, Northern Ireland, Great Britain 2005-2012. Irish Journal of Medical Science / 2014;183(11 SUPPL. 1):S497.	Other reasons
57	Munoz X.; Meca O.; Sanchez-Ortiz M.; Olle-Monge M.; Morell F.; Cruz M.-J. Specific inhalation challenge in occupational asthma:	Other reasons

	Differences in response depending on the type of agent. American Journal of Respiratory and Critical Care Medicine / 2013;187 (MeetingAbstracts).	
58	Mwanga H.H.; Baatjies R.; Singh T.; Jeebhay M. Risk factors for work-related asthma in health care workers with exposure to diverse cleaning agents. Allergy: European Journal of Allergy and Clinical Immunology / 2016;71(Supplement 102):441-442.	Other reasons
59	Nakajima, Tamie; Wang, Hailan; Yuan, Yuan; Ito, Yuki; Naito, Hisao; Kawamoto, Yoshiyuki; Takeda, Kozue; Sakai, Kiyoshi; Zhao, Na; Li, Hongling; Qiu, Xinxiang; Xia, Lihua; Chen, Jiabin; Wu, Qifeng; Li, Laiyu; Huang, Hanlin; Yanagiba, Yukie; Yatsuya, Hiroshi; Kamijima, Michihiro. Increased serum anti-CYP2E1 IgG autoantibody levels may be involved in thepathogenesis of occupational trichloroethylene hypersensitivity syndrome:a case-control study. Arch Toxicol 2022;96(10):2785-2797.	Wrong outcome
60	Ndlela N.H.; Naidoo R.N. Job and exposure intensity among hospital cleaning staff adversely affects respiratory health. American journal of industrial medicine / 2023;((Ndlela) Occupational Health and Safety. United States NLM (Medline) 2023. Not further specified.	Wrong outcome
61	Ngajilo, Dorothy; Singh, Tanusha; Ratshikhopha, Edith; Dayal, Payal; Matuka, Onnicah; Baatjies, Roslynn; Jeebhay, Mohamed F. Risk factors associated with allergic sensitization and asthma phenotypes among poultry farm workers. American Journal of Industrial Medicine 2018;61(6):515-523.	Wrong exposure
62	Nieuwenhuizen, Natalie E. Anisakis - immunology of a foodborne parasitosis. Parasite immunology 2016;38(9):548-557.	Other reasons
63	Nynäs, Pia; Vilpas, Sarkku; Kankare, Elina; Karjalainen, Jussi; Lehtimäki, Lauri; Numminen, Jura; Tikkakoski, Antti; Kleemola, Leenamaija; Huhtala, Heini; Uitti, Jukka. Multiple Chemical Sensitivity in Patients Exposed to Moisture Damage atWork and in General Working-Age Population-The SAMDAW Study. Int J Environ Res Public Health 2021;18(23).	No association
64	Pacheco Da Silva E.; Ngutuka M.; Dumas O.; Orsi L.; Ait-Hadad W.; Lemire P.; Quentin J.; Pin I.; Varraso R.; Siroux V.; Le Moual N. Longitudinal associations of household use of cleaning agents and asthma symptoms in women: the EGEA study. Occupational and environmental medicine / 2023.	Wrong exposure
65	Park J.-H.; Cox-Ganser J.M.; White S.K.; Laney A.S.; Caulfield S.M.; Turner W.A.; Sumner A.D.; Kreiss K. Bacteria in a water-damaged building: associations of actinomycetes and non-tuberculous mycobacteria with respiratory health in occupants. Indoor air / 2017;27(1):24-33.	Wrong exposure
66	Pelta Fernandez, Roberto; De Miguel Diez, Javier; Alvarez-Perea, Alberto; Magan Tapia, Purificacion; Jimenez Garcia, Rodrigo; De Burgoa Gomez-Pinan, Veronica Sanz. Risk Factors for Asthma Onset Between the Ages of 12 and 40. Results of the FENASMA Study. Archivos de Bronconeumologia 2011;47(9):433-440.	Wrong exposure

67	Popescu F.-D.; Vieru M.; Ganea C.S. Allergy risk of exposure to circus tigers in a cat-allergic patient. Allergy: European Journal of Allergy and Clinical Immunology / 2015;70(SUPPL. 101):326.	Other reasons
68	Puvvula J.; Baccaglini L.; Johnson A.; Du Y.; Bell J.E.; Rautiainen R.H. Prevalence and Risk Factors for Pulmonary Conditions among Farmers and Ranchers in the Central United States. Journal of agromedicine / 2022;27(4):378-390.	Wrong outcome
69	Qorbani, Mostafa; Yunesian, Masud. Solid fuel smoke exposure and risk of obstructive airways disease. Iranian Journal of Environmental Health Science & Engineering 2012;9(Journal Article):8-8.	Wrong exposure
70	Quinot C.; Siroux V.; Temam S.; Demange V.; Dananche B.; Varraso R.; Le Moual N.; Dumas O. Occupational exposure and asthma control: A longitudinal analysis controlling for the healthy worker effect. Occupational and Environmental Medicine / 2018;75(Supplement 1):A23-A24.	Other reasons
71	Rajanayagam N. The prevalence of respiratory and skin disease in spray painters. Internal Medicine Journal / 2014;44(Supplement 3):23.	Other reasons
72	Rask-Andersen A. Years in farming, inhalation fever (ODTS) and smoking increases the risk for asthma and other respiratory symptoms in farmers. European Respiratory Journal / 2016;48(Supplement 60).	Other reasons
73	Raulf, M.; Bruening, T.; van Kampen, V. Occupational allergies: to what extent do gender aspects play a role? Allergologie 2017;40(3):117-127.	Wrong exposure
74	Raulf, Monika; Bruening, Thomas; Jensen-Jarolim, Erika; van Kampen, Vera. Gender-related aspects in occupational allergies - Secondary publication and update. World Allergy Organization Journal 2017;10(Journal Article):44-44.	Other reasons
75	Reis L.V.T.D.; Bastos V.P.; Castro M.C.S.D.; Chauvet P.R.; Bartholo T.P.; Lopes A.J.; Pinto B.M.; Faria L.F.; Silva R.V.D.; Rufino R.L.; Costa C.H. Prevalence of patients exposed to sensitizing agents causing occupational asthma. American Journal of Respiratory and Critical Care Medicine / 2017;195.	Other reasons
76	Remen, T.; Acouetey, D-S; Paris, C.; Hannhart, B.; Poussel, M.; Chenuel, B.; Barbaud, A.; Zmirou-Navier, D. Early incidence of occupational asthma is not accelerated by atopy in the bakery/pastry and hairdressing sectors. International Journal of Tuberculosis and Lung Disease 2013;17(7):973-981.	Wrong exposure
77	Remen, Thomas; Acouetey, Dovi-Stephanie; Paris, Christophe; Zmirou-Navier, Denis. Diet, occupational exposure and early asthma incidence among bakers, pastry makers and hairdressers. Bmc Public Health 2012;12(Journal Article):387-387.	Wrong exposure
78	Rollins, Steven M; Su, Feng-Chiao; Liang, Xiaoming; Humann, Michael J; Stefaniak, Aleksandr B; LeBouf, Ryan F; Stanton, Marcia L; Virji, Mohammed A; Henneberger, Paul K. Workplace indoor environmental quality and asthma-related outcomes in healthcare workers. Am J Ind Med 2020;63(5):417-428.	Wrong exposure
79	Ronmark, Erik P.; Ekerljung, Linda; Mincheva, Roxana; Sjolander, Sigrid; Hagstad, Stig; Wennergren, Goran; Ronmark, Eva; Lotvall, Jan;	Wrong exposure

	Lundback, Bo. Different risk factor patterns for adult asthma, rhinitis and eczema: results from West Sweden Asthma Study. Clinical and Translational Allergy 2016;6(Journal Article):28-28.	
80	Rosenman K.D.; Millerick-May M.; Reilly M.J.; Flattery J.; Weinberg J.; Harrison R.; Lumia M.; Stephens A.C.; Borjan M. Swimming facilities and work-related asthma. Journal of Asthma / 2015;52(1):52-58.	Wrong outcome
81	Runeson-Broberg R.; Norback D. Work-related psychosocial stress as a risk factor for asthma, allergy, and respiratory infections in the Swedish workforce. Psychological Reports / 2014;114(2):377-389.	No association
82	Sander, Ingrid; Rihs, Hans-Peter; Doekes, Gert; Quirce, Santiago; Krop, Esmeralda; Rozynek, Peter; van Kampen, Vera; Merget, Rolf; Meurer, Ursula; Bruening, Thomas; Raulf, Monika. Component-resolved diagnosis of baker's allergy based on specific IgE to recombinant wheat flour proteins. Journal of Allergy and Clinical Immunology 2015;135(6):1529-1537.	No association
83	Sander, Ingrid; Rihs, Hans-Peter; Doekes, Gert; Quirce, Santiago; Krop, Esmeralda; Rozynek, Peter; van Kampen, Vera; Merget, Rolf; Meurer, Ursula; Bruening, Thomas; Raulf, Monika. Component-resolved diagnosis of baker's allergy based on specific IgE to recombinant wheat flour proteins. Journal of Allergy and Clinical Immunology 2015;135(6):1529-1537.	No association
84	Saricaoglu, Hayriye; Toka, Sevil Ovali; Algan, Sema Ipek. Latex allergy in health care workers. Turkderm-Archives of the Turkish Dermatology and Venerology 2013;47(2):94-98.	Wrong outcome
85	Sava F.; Gautrin D.; Cartier A.; Lemiere C. Sensitization to detergent enzymes in healthcare workers. American Journal of Respiratory and Critical Care Medicine / 2014;189(MeetingAbstracts).	Other reasons
86	Schantora, A. L.; Casjens, S.; Deckert, A.; van Kampen, V.; Neumann, H. -D; Bruening, T.; Raulf, M.; Buenger, J.; Hoffmeyer, F. Prevalence of Work-Related Rhino-Conjunctivitis and Respiratory Symptoms Among Domestic Waste Collectors. Environment Exposure to Pollutants 2015;834(Journal Article):53-61.	Wrong exposure
87	Schyllert C.; Hedlund U.; Hedman L.; Ronnmark E.; Lindberg A. Occupational airborne exposure to chemicals increase the risk for asthma and rhinitis. European Respiratory Journal / 2014;44(SUPPL. 58).	Other reasons
88	Schyllert C.; Hedman L.; Andersson M.; Hedlund U.; Lundback B.; Ronmark E.; Lindberg A. Increased risk of asthma and rhinitis after exposure to chemicals. European Respiratory Journal / 2015;46(SUPPL. 59).	Other reasons
89	Schyllert, Christian; Ronmark, Eva; Andersson, Martin; Hedlund, Ulf; Lundback, Bo; Hedman, Linnea; Lindberg, Anne. Occupational exposure to chemicals drives the increased risk of asthma and rhinitis observed for exposure to vapours, gas, dust and fumes: a cross-sectional population-based study. Occupational and environmental medicine 2016;73(10):663-669.	Wrong exposure

90	Sejbaek, Camilla Sandal; Flachs, Esben Meulengracht; Carøe, Tanja Korfitsen; Meye, Harald William; Frederiksen, Marie; Frydendall, Karen Bo; Wolkoff, Peder; Clausen, Per Axel; Hougaard, Karin Sørig; Schlünssen, Vivi. Professional cleaning and risk of asthma - a Danish nationwideregister-based study. Scand J Work Environ Health 2021;48(2):127-136.	Wrong exposure
91	Sejbaek C.S.; Flachs E.M.; Caroe T.K.; Meyer H.W.; Frederiksen M.; Frydendall K.B.; Wolkoff P.; Clausen P.A.; Hougaard K.S.; Schlunssen V. Professional cleaning and risk of asthma - a Danish nationwide register-based study. Scandinavian Journal of Work, Environment and Health / 2022;48(2):127-136.	Wrong exposure
92	Seok, Hongdeok; Yoon, Jin-Ha; Won, Jong-Uk; Lee, Wanhyung; Lee, June-Hee; Jung, Pil Kyun; Roh, Jaehoon. Concealing Emotions at Work Is Associated with Allergic Rhinitis in Korea. Tohoku Journal of Experimental Medicine 2016;238(1):25-32.	Wrong exposure
93	Sharifi, Laleh; Karimi, Akram; Shoormasti, Raheleh Shokouhi; Miri, Sara; Nazhad, Hassan Heydar; Bokaie, Saied; Fazlollahi, Mohammad Reza; Haghighi, Khosro Sadeghnhat; Pourpak, Zahra; Moin, Mostafa. Asthma Symptoms and Specific IgE Levels among Toluene Diisocyanate (TDI) Exposed Workers in Tehran, Iran. Iranian Journal of Public Health 2013;42(4):397-401.	Wrong exposure
94	Sherson, D.; Nielsen, A. D.; Mortz, C. G.; Vestergaard, L.; Brandt, L. P. A.; Jors, E.; Baelum, J. Occupational rhinoconjunctivitis caused by the common indoor plant, Hoya compacta. Occupational Medicine-Oxford 2017;67(6):490-492.	Wrong outcome
95	Shiryaeva, Olga; Aasmoe, Lisbeth; Straume, Bjorn; Bang, Berit Elisabeth. Respiratory symptoms, lung functions, and exhaled nitric oxide (FENO) in two types of fish processing workers: Russian trawler fishermen and Norwegian salmon industry workers. International Journal of Occupational and Environmental Health 2015;21(1):53-60.	No association
96	Sit, Guillaume; Letellier, Noémie; Iwatsubo, Yuriko; Goldberg, Marcel; Leynaert, Bénédicte; Nadif, Rachel; Ribet, Céline; Roche, Nicolas; Roquelaure, Yves; Varraso, Raphaëlle; Zins, Marie; Descatha, Alexis; Le Moual, Nicole; Dumas, Orianne. Occupational Exposures to Organic Solvents and Asthma Symptoms in the CONSTANCES Cohort. Int J Environ Res Public Health 2021;18(17).	Wrong exposure
97	Soyseth, Vidar; Johnsen, Helle Laier; Henneberger, Paul K.; Kongerud, Johny. The Incidence of Work-related Asthma-like Symptoms and Dust Exposure in Norwegian Smelters. American Journal of Respiratory and Critical Care Medicine 2012;185(12):1280-1285.	Wrong exposure
98	Steinemann, Anne. Chemical sensitivity, asthma, and effects from fragranced consumer products: national population study in Sweden. Air Quality Atmosphere and Health 2019;12(2):129-136.	Wrong exposure
99	Stoecklin-Marois, Maria T.; Bigham, Corina W.; Bennett, Deborah; Tancredi, Daniel J.; Schenker, Marc B. Occupational Exposures and Migration Factors Associated With Respiratory Health in California Latino Farm Workers The MICASA Study. Journal of Occupational and Environmental Medicine 2015;57(2):152-158.	Wrong outcome

100	Stoeva, Iliyana; Dencheva, Maria; Mircheva, Kristina; Chonin, Atanas. Respiratory Symptoms of Exposure to Substances in the Workplace among Bulgarian Dental Students: a Self-report Questionnaire Survey. Folia Med (Plovdiv) 2020;62(1):141-146.	Wrong population
101	Stoeva, Iliyana; Dencheva, Maria; Mircheva, Kristina; Chonin, Atanas. Respiratory Symptoms of Exposure to Substances in the Workplace among Bulgarian Dental Students: a Self-report Questionnaire Survey. Folia Med (Plovdiv) 2020;62(1):141-146.	Wrong population
102	Stoeva, I. Respiratory symptoms of exposure to substances in the workplace among dental laboratory technicians. Medycyna pracy 2021;72(2):105-111.	Wrong outcome
103	Straumfors, Anne; Eduard, Wijnand; Andresen, Knut; Sjaastad, Ann Kristin. Predictors for Increased and Reduced Rat and Mouse Allergen Exposure in Laboratory Animal Facilities. Annals of Work Exposures and Health 2018;62(8):953-965.	Wrong outcome
104	Suojalehto H.; Suuronen K.; Cullinan P.; Lindstrom I.; Sastre J.; Walusiak-Skorupa J.; Munoz X.; Talini D.; Klusackova P.; Moore V.; Merget R.; Svanes C.; Mason P.; dell'Omo M.; Moscato G.; Quirce S.; Hoyle J.; Sherson D.; Preisser A.; Seed M.; Rifflart C.; Godet J.; de Blay F.; Vandenplas O. Phenotyping occupational asthma caused by acrylates in a multicentre cohort study. The journal of allergy and clinical immunology. In practice / 2019.	Other reasons
105	Supapvanich, Chompunuch; Povey, Andrew C.; de Vocht, Frank. Latex sensitization and risk factors in female nurses in Thai governmental hospitals. International journal of occupational medicine and environmental health 2014;27(1):93-103.	Wrong exposure
106	Svanes O.; Skorge T.D.; Forsberg B.; Gislason T.; Holm M.; Janson C.; Johannessen A.; Jogi R.; Lygre S.H.; Macsali F.; Norback D.; Omenaas E.; Real F.G.; Schlunssen V.; Sigsgaard T.I.; Toren K.; Wieslander G.; Aasen T.; Svanes C. Asthma and COPD in cleaners from Northern Europe. European Respiratory Journal / 2013;42(SUPPL. 57).	Other reasons
107	Szeszenia-Dabrowska, Neonila; Swiatkowska, Beata; Wilczynska, Urszula. Occupational Diseases among Farmers in Poland. Medycyna pracy 2016;67(2):163-171.	Wrong exposure
108	Tafuro, Federica; Ridolo, Erminia; Goldoni, Matteo; Montagni, Marcello; Mutti, Antonio; Corradi, Massimo. Work-related allergies to storage mites in Parma (Italy) ham workers. Bmj Open 2015;5(5):e007502-e007502.	Wrong exposure
109	Tagiyeva, Nara; Teo, Edmund; Fielding, Shona; Devereux, Graham; Semple, Sean; Douglas, Graham. Occupational exposure to asthmagens and adult onset wheeze and lung function in people who did not have childhood wheeze: A 50-year cohort study. Environment international 2016;94(Journal Article):60-68.	Wrong exposure
110	Talini, Donatella; Ciberti, Alessandro; Bartoli, Dusca; Del Guerra, Paolo; Iaia, Tonina Enza; Lemmi, Maria; Innocenti, Andrea; Di Pede, Francesco; Latorre, Manuela; Carrozzi, Laura; Paggiaro, Pierluigi. Work-related asthma in a sample of subjects with established asthma. Respiratory medicine 2017;130(Journal Article):85-91.	Wrong exposure

111	Tarigan, Yenni Gustiani; Chen, Ruey-Yu; Lin, Hsiu-Chen; Jung, Chia-Yi; Kallawicha, Kraiwuth; Chang, Ta-Pang; Hung, Po-Chen; Chen, Chih-Yong; Chao, Hsing Jasmine. Fungal Bioaerosol Exposure and its Effects on the Health of Mushroom and Vegetable Farm Workers in Taiwan. Aerosol and Air Quality Research 2017;17(8):2064-2075.	Other reasons
112	Thanasias, E.; Polychronakis, I.; van Kampen, V.; Bruening, T.; Merget, R. Occupational Immediate-Type Allergic Asthma due to Potassium Tetrachloroplatinate in Production of Cytotoxic Drugs. Respiratory Regulation - Clinical Advances 2013;755(Journal Article):47-53.	Wrong exposure
113	Toletone, Alessandra; Dini, Guglielmo; Massa, Emanuela; Bragazzi, Nicola Luigi; Pignatti, Patrizia; Voltolini, Susanna; Durando, Paolo. Chlorhexidine-induced anaphylaxis occurring in the workplace in a health-care worker: case report and review of the literature. Medicina Del Lavoro 2018;109(1):68-76.	Wrong outcome
114	Toren, Kjell; Ekerljung, Linda; Kim, Jeong-Lim; Hillstrom, Jenny; Wennergren, Goran; Ronmark, Eva; Lotvall, Jan; Lundback, Bo. Adult-onset asthma in west Sweden - Incidence, sex differences and impact of occupational exposures. Respiratory medicine 2011;105(11):1622-1628.	Wrong exposure
115	Tsui H.-C.; Ronsmans S.; Hoet P.H.M.; Nemery B.; Vanoirbeek J.A.J. Occupational Asthma Caused by Low-Molecular-Weight Chemicals Associated With Contact Dermatitis: A Retrospective Study. Journal of Allergy and Clinical Immunology: In Practice / 2022;10(9):2346-2354.e4.	Other reasons
116	Yong, Mei; Morfeld, Peter; McCunney, Robert. Extended Investigation of Exposure to Respirable Synthetic AmorphousSilica Dust and Its Potential Impact on Non-malignant Respiratory Morbidity. Front Public Health 2022;10():801619.	Wrong outcome
117	Tynes T.; Lovseth E.K.; Johannessen H.A.; Sterud T.; Skogstad M. Interaction of smoking with respiratory effects of occupational dust exposure: A prospective population study among Norwegian men. ERJ Open Research / 2018;4(2):00021-2018.	Other reasons
118	Utsugi, Harue; Usui, Yutaka; Nishihara, Fuyumi; Kanazawa, Minoru; Nagata, Makoto. Mycobacterium gordonae-induced humidifier lung. Bmc Pulmonary Medicine 2015;15(Journal Article):108-108.	Other reasons
119	van der Walt, Anita; Singh, Tanusha; Baatjies, Roslynn; Lopata, Andreas Ludwig; Jeebhay, Mohamed Fareed. Work-related allergic respiratory disease and asthma in spice mill workers is associated with inhalant chili pepper and garlic exposures. Occupational and environmental medicine 2013;70(7):446-452.	Wrong exposure
120	van Rooy, F. G. B. G. J.; Houba, R.; Stigter, H.; Zaat, V. A. C.; Zengeni, M. M.; Rooyackers, J. M.; Boers, H. E.; Heederik, D. J. J. A cross-sectional study of exposures, lung function and respiratory symptoms among aluminium cast-house workers. Occupational and environmental medicine 2011;68(12):876-882.	Wrong exposure

121	Vandenplas O.; D'Alpaos V.; Evrard G.; Huaux F.; Thimpont J. Occupational asthma due to cleaning agents. European Respiratory Journal / 2013;42(SUPPL. 57).	Other reasons
122	Vandenplas O.; Godet J.; Hurdubaea L.; Rifflart C.; Suojalehto H.; Wiszniewska M.; Munoz X.; Sastre J.; Klusackova P.; Moore V.; Merget R.; Talini D.; Svanes C.; Mason P.; dell'Omo M.; Cullinan P.; Moscato G.; Quirce S.; Hoyle J.; Sherson D.L.; Kauppi P.; Preisser A.; Meyer N.; de Blay F.; Pirjo H.; Patrizia P.; Giann P.; Carolina B.; Pierluigi P.; Ilenia F.; Jorunn K.; Jolanta W.-S.; Christian R.-M.; Mar F.-N.; Gemma V.-N. Are high- and low-molecular-weight sensitizing agents associated with different clinical phenotypes of occupational asthma? Allergy: European Journal of Allergy and Clinical Immunology / 2019;74(2):261-272.	No association
123	Vu, Mi; Bala, Harini Rajgopal; Cahill, Jennifer; Toholka, Ryan; Nixon, Rosemary. Immediate hypersensitivity to chlorhexidine. Australasian Journal of Dermatology 2018;59(1):55-56.	Wrong outcome
124	Walters G.I.; Kirkham A.; McGrath E.E.; Moore V.C.; Robertson A.S.; Burge P.S. 21 years of SHIELD: Decreasing incidence of Occupational Asthma in the West Midlands, UK? Thorax / 2013;68(SUPPL. 3):A168-A169.	Other reasons
125	Walters G.; Robertson A.; Moore V.; Burge S. Occupational asthma from sensitization to a chlorine-containing triclosan cleaner. European Respiratory Journal / 2014;44(SUPPL. 58).	Other reasons
126	Wang L.; Rosenman K. Adverse Health Outcomes Among Industrial and Occupational Sectors in Michigan. Preventing chronic disease / 2018;15((Rosenman) Department of Medicine, Michigan State University, East Lansing, MI, United States):E102.	Wrong outcome
127	Watanabe M.; Kurai J.; Sano H.; Torai S.; Yanase H.; Funakoshi T.; Fukada A.; Hayakawa S.; Kitano H.; Shimizu E. Prevalence of allergic rhinitis based on the SACRA questionnaire among Japanese nursing professionals with asthma. Journal of Medical Investigation / 2016;63(1-2):108-113.	Other reasons
128	Weinmann T.; Gerlich J.; Heinrich S.; Nowak D.; Von Mutius E.; Vogelberg C.; Roller D.; Genuneit J.; AlKhadra S.; Lanzinger S.; Lohse T.; Motoc I.; Walter V.; Weinmayr G.; Radon K. To spray or not to spray? The association of household cleaning agents and disinfectants with asthma in young adults - Results from a crosssectional analysis in Germany. European Respiratory Journal / 2015;46(SUPPL. 59).	Other reasons
129	White G.E.; Seaman C.; Filios M.S.; Mazurek J.M.; Flattery J.; Harrison R.J.; Reilly M.J.; Rosenman K.D.; Lumia M.E.; Stephens A.C.; Pechter E.; Fitzsimmons K.; Davis L.K. Gender differences in work-related asthma: Surveillance data from California, Massachusetts, Michigan, and New Jersey, 1993-2008. Journal of Asthma / 2014;51(7):691-702.	No association
130	White, Gretchen E.; Mazurek, Jacek M.; Storey, Eileen. Employed adults with asthma who have frequent workplace exposures. Journal of Asthma 2015;52(1):46-51.	Wrong exposure

131	Zhang M.; Wang X.F.; Cui X.M.; Wang J.; Yu S.X. The Relationship between Working Conditions and Adverse Health Symptoms of Employee in Solar Greenhouse. Biomedical and environmental sciences: BES / 2015;28(2):143-147.	Other reasons

Appendix 7. Study characteristics and risk of bias of the 55 included studies published between 2011 and 2023

**Author**	**Study design**	**Population**	Participation **rate**	Exposure		Outcome	Confounders adjusted for	**Exposure-response analysis**	**Confidence in study results**
				Definition	Assessment	Assessment			
	**1**	**2**	3	4	5, 6	7	8	9	10
Al-Abcha, 2020(13)	Case-series	Male and female workers aged 20-59 years using or manufacturing carbide tools (N=35)	100 %	*Metals*: Cobalt	Self-reported job history (interview) and workplace enforcement inspections	Physician diagnosed asthma based on symptoms and lung function tests	Self-control	No	
Risk of bias	0	0	0	1	0, 1	1	0	0	Low
Baur, 2013(14)	Case-report	60-year old male chemical worker in the production and packaging of detergents for 32 years (N=1)	100 %	*Enzymes:* Bacterial alpha-amylase termamyl	Self-report and expert assessment	History of work-related asthma symptoms, lung-function test (FEV_1/FVC with or without bronchodilator), IgE measurement (Savinase, Termamyl, alkalase, cellulase, fungal alpha-amylase, total), SPT (common allergens)	Self-control	No	
Risk of bias	0	0	0	1	0, 1	1	0	0	Low
Beach, 2012(15)	Cohort	Male and females with a claim to Workers' Compensation Board (N=11 486)	83 %	*Different exposures:* 8 HMW agents, 4 LMW agents, 3 mixed agents (e.g., animal antigens, cleaning products, shellfish, latex, metal and fumes, metal working fluids)	Register information on occupational code combined with an expert-based JEM	Physician billing for asthma (ICD-9 493) 12 months before a Workers' Compensation Board claim without asthma previous years	Cases and referents matched by age, date of the case claim, prior number of claims, sex	No	
Risk of bias	0	1	1	0	1, 0	0	0	0	Low
Bertelsen, 2016(16)	Case-report	48-year old female worker in a plant producing marine savory seafood ingredients (N=1)	100 %	*Crustaceans:* Shellfish powder (shrimp)	Self-report and expert assessment. Non-blinded SIC; control exposure lactose powder (placebo), active exposure shellfish powder from the plant	History of work-related asthma symptoms, lung function tests (FEV_1, FVC, FEV_1/FVC, PEF, FeNO, DLCO, methacholine provocation test), IS, IgA, IgE, IgM, IgG measurements (food allergens, shrimp, total), IgE for common allergens (Phadiatop), total IgE	Self-control	Yes	
Risk of bias	1	0	0	1	0, 1	1	1	1	High
Branicka, 2021(17)	Case-report	32-year old female oyster mushroom farmer (N=1)	100 %	*Mushrooms*: Oyster mushroom	Self-report and prick-to-prick test with oyster mushroom	Lung function test (FEV1, FVC, PEF), SPT (inhalant and food allergens), IgE measurements	Self-control	No	

Risk of bias	0	0	0	1	0, 1	1	0	0	Low
Brooks, 2020(18)	CS	Male and female cleaners employed at hospitals, schools, and industrial settings (N=425). References comprised retail/service workers and bus drivers (N=281)	Cleaners=74% References= 34%	*Other chemicals*: Cleaning products	Self-report	Questionnaire based interview, lung function test (pre- and post-bronchodilator measurements, FEV1, and FVC), and SPT	Age, ethnicity, asthma medication, and smoking	No	
Risk of bias	0	0	0	0	0, 1	1	0	0	Low
Carder, 2019(19)	Case-series	Male and female cases of work-related respiratory diseases from three surveillance schemes 1989-2017: SWORD (67% females, mean age 43), OPRA (70 % females, mean age 43), and THOR-GP (56% males, mean age 44) (N=779)	100 %	*Other chemicals*: Cleaning agents (e.g., chlorhexidine, formaldehyde, glutaraldehyde, xylenon etc.)	Cases were screened on the likelihood of use of cleaning agents based on previous information recorded by physicians and the recorded occupation and industry	Physician-diagnosed asthma	Self-control	No	
Risk of bias	0	0	0	1	1, 1	0	0	0	Low
Cha, 2012(20)	CS	Male and female self-employed farmers near an oil spill (N=2882)	NS	*Pesticide:* Paraquat (1,1'-dimethyl-4,4' bipyridinium dichloride)	Interview	Questionnaire: Asthma defined in terms of the subject having ever been diagnosed with the disease by a physician	Age, alcohol, education, cumulative exposure of three pesticides, distance from oil spill site, sex, smoking	No	
Risk of bias	0	0	0	1	0, 1	0	0	0	Low
Dumas, 2014(21)	CS	Estonian male and female workers aged 18-65 from population-based biobank, patients, and volunteers from media campaign (N=34 015)	88.3 %	*Different:* 18 known asthmagens e.g., animals, enzymes, latex, highly reactive chemicals, cleaning/disinfecting products, metals	Longest held job combined with a asthma-specific JEM	Interviews by medical personnel: "Do you have asthma now", asthma confirmed by physician, health status and medication	Age, sex, smoking	No	
Risk of bias	0	0	1	0	1, 0	0	0	0	Low
Dumas, 2020(22)	Cohort	Female nurses aged 44 to 68 years,	52.8 %	*Other chemicals:* Disinfectants/cleaning products (e.g., ethylene	Questionnaire and a Job-Task-Exposure matrix	Questionnaire: Self-reported diagnose of asthma and the use of asthma medication	Age, calendar year, race, ethnicity,	No	

		participating in the Nurses' Health Study II (N=61.539)		oxide, hydrogen peroxide, orthophtalaldehyde, formaldehyde, glutaraldehyde, hypochlorite bleach, alcohol, quaternary ammonium compounds, and enzymatic cleaners)			smoking, BMI, and menopausal status		
Risk of bias	0	1	0	1	0, 0	0	0	0	Low
Dumas, 2021(23)	Cohort	Female nurses aging 20 to 52 years, participating in the Nurses' Health Study III (N=17.280)	63%	*Other chemicals:* High-level disinfectants (e.g., aldehydes, hydrogen peroxide)	Self-reported exposure to high-level disinfectants from questionnaires	Questionnaire: Self-reported clinician-diagnosed asthma	Age, race, ethnicity, smoking, and BMI	No	
Risk of Bias	0	1	1	0	0, 1	0	0	1	Moderate
Fishwick, 2022(24)	CS	Pesticide applicators with a mean age of 54.1 years, consisting of 98% males (N=2578)	54%	*Other chemicals:* Pesticides (herbicides, fungicides, and insecticides)	Self-reported exposure to pesticides from a questionnaire	Questionnaire: Self-reported physician-diagnosed asthma: "Has a doctor ever told you that you have asthma?"	Age, sex, and smoking	Yes	
Risk of bias	0	0	0	0	0, 1	0	0	1	Low
Ghosh, 2013(25)	Cohort	Male and females who participated in the National Child Development Study, born 3-9 March 1958 (N=7406)	NS	*Different:* 18 high-risk workplace substances; animal antigens, shellfish, antigenic enzymes, highly reactive chemicals, cleaning products, metal and metal fume antigens, reactive chemicals, cleaning products, metal fumes	Self-reported job history (interview) combined with an asthma-specific expert-based JEM	At age 44-45: Lung-function test (FEV_1/FVC), IgE (total, dust, cat, grass). Interview: Self-reported adult-onset asthma, self-reported adult asthma and self-reported adult asthma with obstruction (FEV_1/FVC<70).	Father's social class at birth, hay fever, region, sex	No	
Risk of bias	0	1	0	0	1, 0	1	0	0	Low
Gonzalez, 2014(26)	CS	Male and female healthcare workers aged 18-65 of years i.e., physicians, nurses, cleaners, radiological technicians, physiotherapist, administrative personnel (N=543)	77 %	*Other chemicals:* Cleaning products i.e., chlorinated/bleach, cleaning/disinfection-related chemicals, glutaraldehyde, latex gloves, quaternary ammonium compounds	Questionnaire, material data sheets, workplace observations	Self-reported physician diagnosed asthma ("Have you ever had asthma" and "Was it confirmed by a doctor"), respiratory symptoms, IgE measurements (e.g., latex, quaternary ammonium compounds)	Age, atopy, BMI, chlorinated/bleach, latex gloves, sex, smoking, quaternary ammonium compounds	No	
Risk of bias	0	0	1	1	0, 1	0	1	0	Moderate

Helaskoski, 2014(27)	Case-series	Male and female patients (25-52 years of age) from occupational medicine clinic (N=5)	100 %	*Highly reactive chemicals:* Hair dressing products e.g., persulfates, permanent wave solutions, hair bleach	Questionnaire, SIC (lactose powder, oxidative hair dyes, not blinded)	History of respiratory symptoms, lung-function test (FEV_1, PEF, histamine challenge, peak flow at/off work, FeNO), IgE measurements (total), SPT (common environmental allergens, most common hairdresser chemicals e.g., oxidative hair dyes), open skin testing (hair dye products), patch test (hairdresser chemicals)	Self-control	No	
Risk of bias	1	0	0	1	0, 1	1	0	0	Moderate
Hougaard, 2012(28)	Case-report	18 year-old female hairdressing apprentice; apprenticeship for 2 years (N=1)	100 %	*Other chemicals:* Persulfate salts (potassium persulfate and ammonium persulfate)	Self-report and expert assessment	History of work-related asthma symptoms, lung-function test (daily PEF), SPT (common allergens, hairdressing series i.e., potassium persulfate and ammonium persulfate), patch teat (standard series, hairdressing series)	Self-control	No	
Risk of bias	0	0	0	1	0, 1	1	0	0	Low
Hoy, 2013(29)	Cohort	Male and female school children of 7-10 years of age (N=792)	NS	*Different exposures:* Eighteen different occupational agents e.g. latex, shellfish, enzymes, highly reactive chemicals, industrial cleaning agents, metal sensitizers, metal working fluids	Self-reported job history combined with an asthma-specific JEM	Questionnaire: Asthma at the age of 44 defined as "Have you ever in your life suffered from attacks of asthma or wheezy breathing?	Sex, smoking	Yes (latex)	
Risk of bias	0	1	0	0	1, 0	0	0	1	Low
Huang, 2016(30)	CC	Male and female adults with adult-onset asthma enrolled from a general hospital. Controls sampled living in the same residential area (N=1102)	NS	*Metals:* Al, As, Ba, Cd, Co, Cr, Cu, Fe, Mn, Mo, Ni, Pb, Rb, Sb, Se, Sn, Sr, Ti, U, V, W, Zn	Interview, urinary measurements (metals)	History of asthma symptoms, physician-diagnosed asthma, lung-function test (daily spirometry, FEV_1, FEV_1/FVC)	Age and sex-matched, BMI, education, occupational dust, family history of asthma, smoking, pets, flower gardening, physical activity	No	
Risk of bias	0	0	0	1	1, 1	1	1	0	Moderate
Huntley, 2022(31)	Case-series	Male and female cases of occupational asthma caused by office work with a mean age of 48 (N=47)	100 %	*Different exposures:* Cleaning agents, metal working fluid, and pesticides	Work history identified from an Occupational Disease Service database and SIC	Lung function test (PEF evaluated with OASYS, FeNo, FEV1/FVC, bronchial reactivity), IgE	Self-control	No	
Risk of bias	1	0	0	0	1, 1	1	0	0	Moderate
Jungewelter, 2019(32)	Case-report	35-year-old female slaughterhouse worker; patient A (N=1)	100 %	*Mammals:* Raw pork meat and kidney	Self-report and expert assessment. SIC (minced raw pork meat and	History of work-related asthma symptoms, lung-function test (FEV_1, FeNO, PEF, histamine), IgE measurements (pig urine protein),	Self-control	No	

					kidney, handling iceberg lettuce, not blinded)	SPT (raw pork meat, pork kidney, pig dander, common aeroallergens)			
Risk of bias	1	0	0	1	0, 1	1	0	0	Moderate
Lastovkova, 2015(33)	Case series	Male and female patients 33-62 years of age from Czech heat-exchanger production line (N=5)	100 %	*Other chemical*: Potassium aluminium tetrafluoride	Workplace measurement of air concentration, SIC (potassium aluminium tetrafluoride powder from the workplace), provocation at workplace (no blinding or sham)	Lung-function test (FEV_1, PEF, MEF, FeNo, non-specific broncho-provocation with histamine or methacholine, bronchodilatation with salbutamol, total airway restiance), IgE measurements (total)	Self-control	No	
Risk of bias	1	0	0	1	0, 1	1	0	0	Moderate
Lawrence 2022(34)	Cohort	Male and female oil spill response and cleanup workers (N=19.018) with a mean age of 42 with no asthma diagnosis.	NS	*Other chemicals:* Benzene, Toluene, Ethylbenzene, Xylenes, n-Hexane.	Full-shift personal air samples with passive organic vapor dosimeters.	Physician-diagnosed asthma.	Age, sex, race, ethnicity, smoking, previous oil industry experience, highest education, and potential exposure to burning/flaring crude oil.	Yes	
Risk of bias	0	1	0	1	1, 1	0	0	1	Moderate
Le Moual, 2012(35)	CS	Enriched female with asthma recruited from a chest clinics, their relatives, group of population-based subjects (N=683)	41.2 %	*Other chemicals:* 24 domestic cleaning exposures (9 cleaning tasks, 15 cleaning agents)	Self-report (questionnaire), component analysis	Asthma symptoms, lung-function test (FEV_1 methacholin challenge test), IgE measurements (total), SPT (12 allergens)	Age, BMI, education level, occupational exposures, smoking	No	
Risk of bias	0	0	0	0	0, 1	1	0	0	Low
Lillienberg, 2013(36)	Cohort	Male and female random sampled from the general Nordic population from seven geographic centres (born 1945-1973) (N=13 284)	74 %	*Different exposures:* E.g., acrylates, cleaning products, reactive chemicals, metal working fluids	Self-reported job history combined with an asthma-specific expert-based JEM	Questionnaire: "Do you have or have you ever had asthma after the age of 16" and "Have you ever had asthma diagnosed by a physician"	Age, atopy	No	
Risk of bias	0	1	1	0	1, 0	0	0	0	Low
Lillienberg, 2014(37)	Cohort	Male and female random sampled from the general Nordic population	74 %	*Different exposures:* E.g., acrylates, cleaning products, reactive chemicals, metal working	Self-reported job history combined with two asthma-	Questionnaire: "Do you have or have you ever had asthma after the age of 16" and "Have you ever had asthma diagnosed by a physician"	Age, atopy	No	

		from seven geographic centres (born 1945-1973) (N=13 284)		fluids, highly reactive chemicals	specific expert-based JEM				
Risk of bias	0	1	1	0	1, 0	0	0	0	Low
Lipinska-Ojrzanowska, 2013(38)	Case-report	51-year-old female process operator in dishwashing tablets factory for 4 years (N=1)	100 %	*Enzymes:* Savinase	Self-reported exposure to cleaning agents, material data sheets. Single blinded SIC; control exposure talcum, active exposure powder of crushed dishwashing tablets	History of work-related asthma symptoms, lung function tests (FEV1, FVC, FEV1/FVC, PEF, methacholine inhalation challenge test), IgE measurements (savinase, total), SPT (common allergens, alfa-amylase)	Self-control	No	
Risk of bias	1	0	0	1	1, 1	1	1	0	High
Lipinska-Ojrzanowska, 2017(39)	Case-series	Female professional cleaners referred for suspected asthma (N=50)	100 %	*Other chemicals:* Cleaning agents e.g., latex and disinfectants	Self-reported exposure to cleaning agents, material data sheets. SIC: placebo, vinyl gloves, NaCl, latex gloves, cleaning agents e.g., disinfectants,	History of work-related symptoms, lung-function test (FEV_1, FVC FEV_1/FVC, PEF with our without salbutamol /methacholine), IS, IgE measurements (total and specific i.e., latex, cleaning agents), SPT (common allergens, latex, cleaning agents)	Self-control	No	
Risk of bias	1	0	0	1	0, 1	1	1	0	Moderate
Liu, 2019(40)	CS	Population-based sample of plastic film greenhouse workers (N=5420)	92.2 %	*Other chemicals:* Pesticide use; type of green house (vegetables, flowers, poultry, mushroom); foul greenhouse odors	Self-report (interview)	History respiratory symptoms, lung-function test (FEV_1/FVC, postbronchodilator test with salbutamol)	NS	No	
Risk of bias	0	0	1	0	0, 1	1	0	0	Low
Mason, 2020(41)	Case-series	Male and female occupational asthma cases from SWORD working in the UK seafood processing sector (N=58)	100 %	*Different exposures*: Crustaceans (prawns, crab, scampi, shrimp, and scallop), fish (salmon, trout), and other agents	NS	Chest physician-diagnosed asthma	Self-control	No	
Risk of bias	0	0	0	1	1, 1	0	0	0	Low
Mason, 2020(42)	Case-report	47-year-old non-atopic woman working as a spray painter in a tannery for 23 years	100 %	*Other chemicals*: Polyfunctional aziridine	SIC with polyfunctional aziridine (one exposure level) with sham and blinded	Confirmed asthma based on symptoms, lung function test, FeNO, induced sputum collection and SPT	Self-control	No	
Risk of bias	1	0	0	1	1, 1	1	1	0	High

Migueres, 2021(43)	Case-series	Subjects with QAC-induced OA (N=22) 89 subjects with OA caused by other LMW agents.		*Other chemicals:* Quaternary ammonium compounds, metals, metal working fluids, amines, anhydrides, epoxy resin	SIC with a QAC alone. Sham unclear	Confirmed asthma based on symptoms, spirometry, medication	Self-control	Unclear	
Risk of bias	1	0		1	0, 1	1	0	0	Low
Moore, 2017(44)	Case-series	Domestic cleaners and healthcare workers (N=4)	100 %	*Other chemicals:* Chlorine-releasing tablets (i.e., chlorine, urine, mix of chlorine and urine (chloramine)	SIC; neutral detergent solution, chlorine-releasing tablets, Haztabs, urine, mix of chlorine and urine, no sham	Confirmed asthma based on lung-function test (FEV_1 with/ without metacholine, FVC, whole day PEF for four week, FeNO pre and post each challenge test, non-specific bronchoprovocation), IgE measurements (e.g., latex)	Own control	No	
Risk of bias	1	0	0	1	0, 1	1	0	0	Moderate
Oppliger, 2017(45)	Cohort	Laboratory animal workers and students e.g., faculties of medicine, veterinary, medicine and science (N=177)	58,6 %	*Mammals:* Rat and mouse antigens, endotoxin	Questionnaire (interview), personal workplace measurements (airborne dust)	History of symptoms, lung-function test (e.g., FEV_1, FVC, FEV_1/FVC), IgE measurements (total, mouse, rat)	BMI, education, nationality, sex, and smoking	No	
Risk of bias	0	1	0	1	1, 1	1	0	0	Moderate
Pacheco Da Silva, 2022(46)	Cohort	Male and females with a mean age of 46.8 years from a French population-based cohort (N=43 507)	61,0 %	*Other chemicals*: Bleach, ammonia, acids, and solvents, and scented products (e.g., cleaning, fragrances, candles, and air freshener), sprays, homemade products, and disinfection wipes.	Self-report (questionnaire)	Questionnaire: Yes to “Have you ever had asthma?” were considered as “ever asthma” and among these, “current asthma” status was classified if yes to asthma symptoms, asthma attacks or asthma treatment were present in the past 12 months.	Sex, age, smoking, BMI, and educational level.	Yes	
Risk of bias	0	1	1	1	0, 1	0	0	1	Moderate
Patel, 2018(47)	CS	Active primary farm operators; responsible for running the farm (N=11 210)	70.8 %	*Other chemicals:* Pesticides herbicides, phenoxy, 2,4-D, glyphosate, insecticides	Questionnaire and information on active ingredients obtain through product research page	Physician diagnosed asthma and still symptoms (current asthma)	Region, sex	No	
Risk of bias	0	0	1	1	0, 1	0	0	0	Low
Patel, 2020(48)	CS	Male and female certified nurse aids registered by the Texas Department of Aging and Disability Services (N=413)	21.6 %	*Other chemicals*: Endoscopy/glutaraldehyde/ Orthophthalaldeyde, Enzymatic cleaners, Bleach/quaternary compounds, sprays and latex	Expert-rated JEM based on the longest job held	Questionnaire: “Have you ever had asthma?” and “Has it been confirmed by a doctor?” Asthma and allergy symptoms were based on eight items.	Race, atopy, obesity, smoking, and years at job	No	

Risk of bias	0	0	0	1	1, 0	0	0	0	Low
Pravettoni, 2014(49)	Case-report	41-year-old female food industry worker (e.g., packaging of various dried mushrooms) (N=1)	100 %	Mushrooms: shiitake mushrooms (Lentinus edodes)	Self-report and expert assessment	History of work-related asthma symptoms, lung-function tests (FEV_1, FVC with/without bronchodilator (salbutamol)), FeNO, SDS-PAGE, IgE-immunoblotting, IgE measurements (total, molds: Aspergillus fumigatus, Alternaria alternate, Penicillum notatum, Cladosporium herbarum, champignon: Agaricus bisporus), SPT (commercial aeroallergens, food allergens e.g., mushrooms, negative controls)	Self-control	No	
Risk of bias	0	0	0	1	0, 1	1	0	0	Low
Roussel, 2012(50)	CS	Archive workers from 10 archive centres (N=144)	54 %	*Molds fungi or yeast;* specific penicillium (i.e., Cladosporium sphaerospermum, Alternaria alternata, Stachybotrys chartarum, Aspergillus fumigatus)	Measurements; Air and dust samples in the 10 archive centers (e.g., molds), questionnaire	Self-reported physician diagnosed asthma	Age, sex, smoking	No	
Risk of bias	0	0	0	1	1, 1	0	0	0	Low
Simoneti, 2016(51)	CS	Male and female workers or students at two universities dealing/working with/without laboratory animals (N=737)	95 %	*Mammals:* Lab animals (i.e., rat, rabbit, mouse, hamster, guinea-pig)	Self-report, dust samples from work room floor (e.g., mouse and rat allergens)	History of respiratory symptoms, lung-function tests (FEV_1, bronchial challenge test with mannitol), SPT (common allergens, laboratory animals i.e., rat, rabbit, mouse, hamster, guinea pig)	Age, concentration of allergens, daily work hours, exposure years, groups, institution, job category, past exposure, pet ownership, sex, smoking	Yes	
Risk of bias	0	0	1	1	1, 1	1	0	1	High
Simoneti, 2017(52)	CS	Male and female students and employees at 2 universities dealing/working with laboratory animals (N=453)	95 %	*Mammals:* Laboratory animals (rat, rabbit, mouse, hamster, and guinea-pig)	Self-reports	History of respiratory symptoms, lung-function tests (FEV_1, bronchial challenge test with mannitol), SPT (common allergens, laboratory animals i.e., rat, rabbit, mouse, hamster, guinea pig)	Age, daily work hours, exposure years, pet ownership, sex, smoking	Yes	
Risk of bias	0	0	1	1	0, 1	1	0	1	Moderate
Singh, 2013(53)	CS	Male and female dental healthcare workers and students, and non-clinical staff from 5 academic dental	NS	*Other chemicals:* Dental spray, mist or stem, and latex	Self-reports	Questionnaire: "Have you had an attack of asthma in the last 12 months" and "Are you currently taking any medication for asthma", lung-function test (FEV_1, FVC, bronchodilator), IgE measurement	Age, sex, smoking	No	

		institutions (N=454)				(common inhalants, latex, horse radish peroxidase, bromelain)			
Risk of bias	0	0	0	1	0, 1	1	0	0	Low
Sit, 2022(54)	Case-control	Male and female population from a French nutritional cohort study – NutriNet-Santé – with a mean age of 54 years (N=4469)	54.5 %	Other chemicals: Disinfection cleaning products and solvents	Expert-based JEM	Questionnaire: "Have you ever had asthma?" and questions related to symptoms, asthma treatment, and asthma attacks	Sex, age smoking, and BMI	Yes	
Risk of bias	0	0	0	0	1, 0	0	0	1	Low
Song, 2013(55)	Case-report	34-year old male wallpaper manufacturer (N=1)	100 %	*Other chemicals:* Polyvinyl Chloride (PVC) *Metals:* Nickel	PVC and stone powder handled at workplace, no measurements, SIC (PVC, nickel), no blinding or sham	History of respiratory symptoms, lung-function test (FeNo, FEV1, methacholin challenge test, bronchial challenge test; saline, PVC), IgE measurements (total), SPT (common allergens, intradermal test (PVC)), patch test (PVC, zinc oxide, Nickel, chromium), sputum eosinophils	Self-control	No	
Risk of bias	1	0	0	1	0, 1	1	0	0	Moderate
Suojalehto, 2018(56)	Case-reports	Factory workers exposed to 3-(Bromomethyl)-2chloro-4(methylsulfonyl)-benzoic acid office worker at the same factory (N=93)	92 %	*Other chemicals:* 3-(Bromomethyl)-2chloro-4(methylsulfonyl)-benzoic acid (BCMBA)	Questionnaire, interview, dust measurements, observations, SIC (3-(Bromomethyl)-2chloro-4(methylsulfonyl)-benzoic acid, control powder; lactose powder)	History of respiratory symptoms, lung-function test (spirometry; FEV_1, histamine challenge, FeNO), IgE measurements (total), SPT (common allergens, Alternaria alternate, Cladosporium herbarum, 3-(Bromomethyl)-2chloro-4(methylsulfonyl)-benzoic acid), open skin application test (3-(Bromomethyl)-2chloro-4(methylsulfonyl)-benzoic acid)	Self-control	Yes	
Risk of bias	1	0	0	1	0, 1	1	1	1	High
Suojalehto, 2019(57)	Case-reports	Referred patients with suspected asthma and working with epoxy resins or triglycidylether (N=113)	100 %	*Other chemicals:* Epoxy resin, polyamine hardeners, triglycidyl isocyanurate (TGIC)	Work place measurements (airborne polyamines and solvents, amines). SIC (butyl acetate solvent, 1-component, solvent-based paint, lactose. powder, epoxy resin, polyamine hardener, triglycidyl isocyanurate)	Symptoms, lung-function test (FeNO, FEV_1, histamine/methacholine, placebo), SPT (serum albumin-conjugated diglyirid eher of bisphenol A epoxy resin, epoxy components)	Self-control	No	

Risk of bias	1	0	0	1	0, 1	1	1	0	Moderate
Suojalehto, 2020(58)	Case-reports	Patients with acrylate-induced asthma (N=55) compared to LMW agents-induced asthma (N=418) and asthma from isocyanates (N=125	100 %	*Other chemicals*: Acrylates (e.g., cyanoacrylates, methacrylates, and plain acrylates)	SIC with acrylates and a placebo control test without acrylates ingredients	Lung function test (FEV1) and markers of airway inflammation	Self-control		
Risk of bias	1	0	0	1	1, 1	1	1	0	Low
Tustin, 2021(59)	CS	Male and female workers from a company manufacturing countertops (N=64)	NS	*Other chemicals*: epoxy resin, phthalic anhydride (PA)	Occupational hygienist walk-throughs of the plant, 6 measurements of PA	Symptomatic workers: Lung function test (FEV1 and FVC), reversibility testing, medical history, Occupational Asthma Screening Questionnaire	Self-control		
Risk of bias	0	0	0	1	1, 1	1	0	0	Moderate
Vandenplas, 2013(60)	Case series	Cleaners who completed a SIC procedure with cleaning agents in a tertiary center (N=44)	100 %	*Other chemicals:* Cleaning agents e.g., quaternary ammonium compounds, glutar-aldehyde, both of these agents and ethanolamines	Interview, data sheets, experts assessment, SIC; open SIC (control with paint diluent, cleaning products)	Lung-function test (spirometry; FEV_1, sputum eosinophils, histamine bronchial reactivity), sputum cell counts	Self-control	Yes	
Risk of bias	1	0	0	1	0, 1	1	1	1	High
Vincent, 2018(61)	CC	Asthma patients with and without mold sensitization (N=64)	NS	*Mold, fungi, yeast:* Alternaria alternata, Cladosporium herbarum, Aspergillus fumigatus, Penicillium spp, Cladosporium sphaerospermum, Cladosporium cladosporioides, Aspergillus versicolor contamination in dwellings	Questionnaire, mold contamination assessed and measured in main rooms at home	History of respiratory symptoms, lung-function test (FEV_1, FVC, FEV_1/FVC) IgE measurements (total, specific molds), SPT or cellulose acetate membrane precipitin to molds (histamine, codeine, A fumigatus, A alternata, Penicillium chrysogenum, and Cherbarum)	Age, sex, smoking	No	
Risk of bias	0	0	0	1	1, 1	1	0	0	Low
Vizcaya, 2013(62)	CC	Male and female cleaners with and without asthma from 37 cleaning companies (N=95)	49.7 %	*Other chemicals:* Cleaning products e.g., ammonia, bleach, degreasers, drain products, dust mop products, glass cleaners, hydrochloric acid	Interview	History of respiratory symptoms, lung-function test (FeNo, methacholine challenge test, exhaled breath condensate spirometry with or without salbutamol; FEV_1, FVC, FEF25-75%) IgE measurements (common aeroallergens, dust mites, latex)	Age, domestic use of cleaning product, sex, smoking	No	
Risk of bias	0	0	0	1	0, 1	1	0	0	Low

Walters, 2017(63)	Case-series	Male and female workers in a variety of industries e.g. manufacturing, health care, education, hair and beauty, printing (n=20)	100 %	*Other chemicals:* Acrylates, acrylic co-polymers, methyl methacrylates	Medical/hygiene reporting from companies, SIC (specific allergens e.g., acrylates (N=11 % of cases)	History of respiratory symptoms, lung-function test (FEV1, FVC, FeNO, PEF, non-specific bronchial reactivity (N=70 %)), IgE measurements (specific allergens (N=46%)), SPT (e.g., common allergens, acrylates)	Self-control	No	
Risk of bias	1	0	0	1	0, 1	1	0	0	Low
Walters, 2018(64)	Case-series	Clinical patients e.g., lifeguards and swimming pool employee, nurses, healthcare cleaner, healthcare assistants, hard hostess, psychiatric ward housekeeper (N=80)	100 %	*Other chemicals:* Cleaning agents e.g., chloramines, glutaraldehyde, quaternary ammonium compounds	Medical/hygiene reporting from companies, SIC (specific cleaning agents (N=11 % of cases)	History of respiratory symptoms, lung-function test (FEV1, FVC, FeNO, PEF, non-specific bronchial reactivity (N=70 %)), IgE measurements (specific allergens (N=46%)), SPT (e.g., common allergens, acrylates)	Self-control	No	
Risk of bias	1	0	0	1	0, 1	1	0	0	Low
Weinmann, 2017(65)	Cohort	Male and female 19-24 year, population based cohort, two German study centers (N=1695)	22.6 %	*Other chemicals:* Cleaning products (i.e., domestic disinfectants; household sprays).	Questionnaire	Questionnaire: Physician-diagnosed asthma and either wheezing without cold or use of asthma medication within the last 12 mounts	Age, sex, socioeconomic status, smoking, study centre	Yes	
Risk of bias	0	1	0	0	0, 1	0	0	1	Low
Weinmann, 2019(66)	Cohort	Male and female, 19-24 year, currently working in health services or jobs involving cleaning (N=356).	17.3 %	*Other chemicals*: Disinfectants.	Questionnaire.	Questionnaire: Doctor-diagnosed asthma and current asthma.	Age, sex, study center, smoking, and domestic use of disinfectants.	Yes	
Risk of bias	0	1	0	1	0, 1	0	0	1	Moderate
Wittczak, 2013(67)	Case-reports	46-year old female nurse in internal medicine ward, 34-year old female nurse in pediatric nephrology ward, 45 year-old female nurse in cardiology (N=3)	100 %	Other chemicals: Chlorhexidine	Exposure assessment not specified, single-blind, placebo-controlled SIC (linen oil, chlorhexidine)	Lung-function test (spirometry, FEV1, FVC, P20, methacholine challenge test),IgE measurements (total, chlorhexidine, natural rubber latex), SPT (common aeroallergens, chlorhexidine), sputum	Self-control	No	
Risk of bias	1	0	0	1	1, 1	1	1	0	High

BMI; Body Mass Index, CC; Case-control study, CS; Cross-sectional study; DLCO; Carbon monoxide diffusing capacity of the lung, FEV1; Forced expiratory volume in one second, FEF25-75; Forced expiratory flow over the middle half of FVC, FeNO; Fractional exhaled nitric oxide, FVC; Forced vital capacity, HMW; High molecular weight, IgE; Immunoglobulin E, IS; induced sputum, JEM; Job exposure matrix, LMW; Low

molecular weight, MEF; Mean expiratory flow, NS; Not specified, OPRA; Occupational Physicians Reporting Activity, SPT; Skin-prick test, SIC; Specific inhalation challenge, SWORD; Surveillance of Work-related and Occupational Respiratory Disease, THOR-GP; The Health and Occupation Research network in General Practice, WRA; work-related asthma,

Appendix 8. Measure of association and conclusion of the 55 included studies published between 2011 and 2023

Allergens	Author	Design	Measure of association	95% CI	Conclusion	Confidence in study result	Initial level of evidence based on the overview	Current level of evidence based on the RCGP-system
**Anhydrides** (not Phthalic anhydride)							Limited or contradictory evidence	Limited or contradictory evidence
Acid anhydrides	Migueres, 2021(43)	Case series	-	-	Acid anhydrides caused asthma in 9 persons	Low		
**Amines**							No evidence	Very limited or contradictory evidence
Amines	Migueres, 2021(43)	Case series	-	-	Amines caused asthma in 10 persons	Low		
**Biocides**							No evidence	Very limited or contradictory evidence
**Pesticides**							(limited or contradictory)	(moderate)
Any pesticide use	Patel, 2018(47)	CS	POR=1.5	1.1-1.8	Statistically significant association for physician diagnosed current asthma; users vs. non-users	Low		
Glyphosate	Patel, 2018(47)	CS	POR=1.3	0.97-1.8	No statistically significant association for physician diagnosed current asthma	Low		
Herbicides	Patel, 2018(47)	CS	POR=1.3	1.0-1.8	The risk of physician diagnosed current asthma was significantly higher among users of herbicides vs. non-users	Low		
Insecticides	Patel, 2018(47)	CS	POR=2.0	1.2-3.3	Statistically significant association for physician diagnosed current asthma	Low		
Multiple pesticides	Liu, 2019(40)	CS	OR=1.24	1.03–1.49	Use of multiple pesticide associated with self-reported or clinically assessed asthma	Low		
Paraquat (1,1’-dimethyl-4,4’ bipyridinium dichloride)	Cha, 2012(20)	CS	OR=2.18	0.99-4.82	The risks of self-reported physician-diagnosed asthma, was non-significantly increased among paraquat-applying farmers compared with non-paraquat-applying farmers	Low		
Pesticides	Huntley, 2022(31)	Case-series	-	-	Pesticide caused asthma in one person	Moderate		
Phenoxy	Patel, 2018(47)	CS	POR=1.4	0.9-2.2	No statistically significant association for physician diagnosed current asthma	Low		
Work with pesticides (hours)	Fishwick, 2022(24)	CS<br>- Low<br>- Zero<br>- High	 OR=1.00<br>OR=1.26<br>OR=0.76	 -<br>0.92-1.73<br>0.55-1.04	No statistically significant association for self-reported doctor diagnosed asthma and hours working with pesticides	Low		
2,4-D	Patel, 2018(47)	CS	POR=1.3	0.8-2.1	No statistically significant association; physician diagnosed current asthma	Low		

**Crustaceans** (not lobsters, snow crabs)							Limited or contradictory evidence	Moderate evidence
Fish/shellfish	Beach, 2012(15)	CC	OR=2.06	1.26-3.49	Increased risk after Bayesian correction for exposure misclassification; Physician billing for asthma	Low		
Fish/shellfish	Ghosh, 2013(25)	Cohort	IPR=12.5	-	IPR estimated from available data; adult onset asthma with airflow limitation. No conclusion drawn.	Low		
Fish and shellfish	Mason, 2020(41)	Case-series						
		-	IR=70	48.9-91.1	A 24-fold occupational asthma excess in the sea food processing sector compared to all other industries			
		Fish/shell - Others	IR=2.9	2.8-3.1		Low		
Fish/shellfish	Hoy, 2013(29)	Cohort	OR=1.0	0.4-2.2	No statistically significant association for self-reported development of asthma after 13 years of age	Low		
Shellfish powder	Bertelsen, 2016(16)	Case-report	-	-	Shellfish powder can cause non-IgE mediated asthma	High		
**Enzymes** (not a-amylase from *Aspergillus oryzae*, detergent enzymes, Papain, Phytase from *Aspergillus niger*, various enzymes from *Bacillus subtilis* (alcalase, protease, maxatase, maxapem, esperase, cellulase, a-amylase, lipase, subtilisin)							Limited or contradictory evidence	Moderate evidence
Antigenic enzymes	Ghosh, 2013(25)	Cohort	OR=5.97	2.14-16.69	Statistical significant association for adult onset asthma with airflow limitation	Low		
Bacteral alpha-amylase termamyl	Baur, 2013(14)	Case-report	-	-	Bacteral alpha-Amylase termamyl caused asthma in one patient	Low		
Bioaerosol enzymes	Lillienberg, 2014(37)	Cohort	HR=1.3	0.6-3.1	No statistically significant association for new-onset asthma	Low		
Enzymes	Hoy, 2013(29)	Cohort	OR=2.2	0.3-16.0	No statistical significant association for development of self-reported asthma after 13 years of age	Low		
Enzymes	Dumas, 2014(21)	CS	OR=2.14	1.08-4.22	Statistically significant association for physician- diagnosed asthma	Low		
Savinase	Lipinska-Ojrzanowska, 2013(38)	Case-report	-	-	Savinase in dishwashing tablets caused asthma in one patient	High		
**Mammals** (not cows, rats)							No evidence	Limited or contradictory evidence
Animals (not specified)	Dumas, 2014(21)	CS	OR=1.62	1.00-2.60	Statistically significant association for physician- diagnosed asthma	Low		
Animal antigens	Ghosh, 2013(25)	Cohort	OR=1.48	0.55-4.01	No statistically significant association for adult onset asthma with airflow limitation	Low		
Animal antigens (HMW)	Beach, 2012(15)	CC	OR=2.42	1.45-4.30	Increased risk after Bayesian correction for exposure misclassification; Physician billing for asthma	Low		
Animal derived	Hoy, 2013(29)	Cohort	OR=1.0	0.5-1.9	No statistically significant association for development of self-reported asthma after 13 years of age	Low		
Lab animals	Simoneti, 2017(52)	CS				Moderate	(strong)	(strong)
		- Years (1.1-3.0)<br>- Years (3.1-5.0)<br>- Years (≥5.1)	PR=1.46<br>PR=3.29<br>PR=2.16	0.42-5.10<br>1.06-10.14<br>0.65-7.19	Workers exposed to lab animals from 3.1-5.0 years of exposure had increased risk of confirmed asthma compared to individuals exposed up to 1.0 year			
Mouse allergen (continuous)	Simoneti, 2016(51)	CS	RR=1.00	0.99-1.01	No association for BHR-confirmed asthma	High		

Rat or mouse antigens	Oppliger, 2017(45)	Cohort	-	-	Rat or mouse caused asthma in 6 persons	Moderate		
Raw pork meat	Jungewelter, 2019(32)	Case-report	-	-	One patient was diagnosed with occupational asthma from raw pork	Moderate		
**Metals** (not Platinum salts)							No evidence	Limited or contradictory evidence
Metal	Dumas, 2014(21)	CS	OR=0.51	0.22-1.16	No statistically significant association for physician- diagnosed asthma	Low		
Metals	Migueres, 2021(43)	Case series	-	-	Metals induced asthma in 26 persons	Low		
Metal and metal fume	Beach, 2012(15)	CC	OR=1.82	1.34-2.66	Increased risk after Bayesian correction for exposure misclassification; Physician billing for asthma	Low		
Metal and metal fume antigens	Lillienberg, 2014(37)	Cohort	HR=1.3	0.6-2.6	No statistically significant association (note: same cohort as for Lillienberg, 2013 but with another JEM)	Moderate		
Metal and metal fume antigens	Ghosh, 2013(25)	Cohort	OR=2.13	1.05-4.32	Statistically significant association for adult onset asthma with airflow limitation	Low		
Metal sensitizers, fumes	Hoy, 2013(29)	Cohort	OR= 0.8	0.4-1.4	No statistically significant association for development of self-reported asthma after 13 years of age	Low		
Metal working fluids	Beach, 2012[10]	CC	OR=2.29	1.71-3.12	Increased risk after Bayesian correction for exposure misclassification; Physician billing for asthma	Low		
Metal working fluids	Ghosh, 2013(25)	Cohort	OR=1.03	0.54-1.97	No statistically significant association for self-reported physician-diagnosed adult onset asthma	Low		
Metal working fluids	Hoy, 2013(29)	Cohort	OR= 0.9	0.2-2.8	No statistically significant association for development of self-reported asthma after 13 years of age	Low		
Metal working fluids	Huntley, 2022(31)	Case-series	-	-	Metal working fluids caused asthma in 1 person	Moderate		
Metal working fluids	Lillienberg, 2013(36)	Cohort	HR=0.9	0.3-2.6	No statistically significant association for new-onset asthma	Low		
Metal working fluids	Migueres, 2021(43)	Case series	-	-	Metals induced asthma in 10 persons	Low		
Ba	Huang, 2016(30)	CC	OR=0.44	0.27-0.80	Statistical significant inverse association for self-reported asthma	Moderate		
Cd	Huang, 2016(30)	CC	OR=1.69	1.00-2.85	Statistical significant association for self-reported asthma	Moderate		
Cr	Huang, 2016(30)	CC	OR=4.89	3.04-7.89	Statistical significant association for self-reported asthma	Moderate		
Co	Al-abcha, 2021(13)	Case-series	-	-	Michigan's surveillance program identified 35 workers with WRA secondary to cobalt. After review, all individuals were classified as having new onset asthma with exposure to cobalt.	Low		
Cu	Huang, 2016(30)	CC	OR=6.06	3.27-11.21	Statistical significant association for self-reported asthma	Moderate		
Fe	Huang, 2016(30)	CC	OR=0.41	0.26-0.64	Statistical significant inverse association for self-reported asthma	Moderate		
Mn	Huang, 2016(30)	CC	OR=0.23	0.14-0.39	Statistical significant inverse association for self-reported asthma	Moderate		
Mo	Huang, 2016(30)	CC	OR=3.76	2.30-6.16	Statistical significant association for self-reported asthma	Moderate		
Ni	Huang, 2016(30)	CC	OR=0.30	0.22-0.41	Statistical significant inverse association for self-reported asthma	Moderate		
Ni	Song, 2013(55)	Case-report	-	-	Nickel (and polyvinyl chloride) caused asthma in one patient	Moderate		

Pb	Huang, 2016(30)	CC	OR=0.48	0.29-0.80	Statistical significant inverse association for self-reported asthma	Moderate		
Rb	Huang, 2016(30)	CC	OR=0.07	0.03-0.15	Statistical significant inverse association for self-reported asthma	Moderate		
Se	Huang, 2016(30)	CC	OR=9.17	4.16-20.21	Statistical significant association for self-reported asthma	Moderate		
U	Huang, 2016(30)	CC	OR=6.99	4.37-11.19	Statistical significant association for self-reported asthma	Moderate		
Zn	Huang, 2016(30)	CC	OR=0.40	0.24-0.66	Statistical significant inverse association for self-reported asthma	Moderate		
**Moud, fungi and yeast** (not Aspergillus, Cladosporium, Penicllium)							Limited or contradictory evidence	Limited or contradictory evidence
Alternaria alternata	Vincent, 2018(61)	CS	-	-	No association with severe asthma	Low		
Contact with moldy documents	Roussel, 2012(50)	CS	OR=1.8	0.5–6.8	No statistically significant association for self-reported physician diagnosed asthma	Low		
Fungi in archives (>1 CFU/m^3)	Roussel, 2012(50)	CS	OR=0.8	0.3–2.4	No statistically significant association for self-reported physician diagnosed asthma	Low		
Fungi in archives (>170 CFU/m^3)	Roussel, 2012(50)	CS	OR=1.5	0.5-4.8	No statistically significant association for self-reported physician diagnosed asthma	Low		
Oyster mushroom	Branicka 2021(17)	Case-report	-	-	Oyster induced asthma in one person	Low		
Shitake mushrooms	Pravettoni, 2014(49)	Case-report	-	-	Shitake mushrooms induced asthma in 1 person	Low		
Molluscs							Limited or contradictory evidence	Limited or contradictory evidence
**Other chemicals** (not drugs, dyes, biocides, and isocyanates)							Limited or contradictory evidence	Limited or contradictory evidence
Cleaning agents							Moderate	Moderate
Acetic acid	Carder, 2019(19)	Case-series	-	-	Acetic acid caused asthma in 1 person	Low		
Alcohol	Dumas, 2020(22)	Cohort	HR=1.14	0.91-1.42	No statistically association for self-reported asthma	Low		
Alcohol degreaser	Walters, 2018(64)	Case-series	-	-	Alcohol degreaser caused asthma in 1 person	Low		
Ammonia	Vizcaya, 2013(62)		OR=2.7	0.9-8.2	No statistically significant association for clinically assessed asthma	Low		
Ammonia	Brooks, 2020(18)	CS<br>- Internal<br>- External	 OR=1.11<br>OR=1.97	 0.58-2.14<br>0.97-4.01	No significant association were found for an internal comparison with cleaners as well as for the external comparison of service workers and bus drivers in current asthma status.	Low		
Benzalkonium chloride	Carder, 2019(19)	Case-series	-	-	Benzalkonium chloride caused asthma in 3 persons	Low		
Benzalkonium chloride	Lipinska-Ojrzanowska, 2017(39)	Case-series	-	-	Benzalkonium chloride induced asthma in 1 person.	Moderate		
Bleach	Vizcaya, 2013(62)	CC	OR=1.1	0.1-1.1	No statistically significant association for clinically assessed asthma.	Low	(moderate)	(moderate)

Bleach	Brooks 2020(18)	CS<br>- Internal<br>- External	 OR=1.04<br>OR=1.87	 0.67-1.64<br>1.12-3.14	No significant association were found for an internal comparison with cleaners, but a significant association were found comparing with an external population of service workers and bus drivers in current asthma status.	Low	
Bleach (hypochlorite)	Dumas 2020(22)	Cohort	HR=1.07	0.84-1.36	No statistically association for self-reported asthma.	Low	
Bleach/chlorine	Gonzalez, 2014(26)	CS	OR=1.01	0.47-2.18	No statistically significant association for physician-diagnosed asthma	Moderate	
Bleach/quatemary compounds	Patel, 2020(48)	CS	OR=0.70	0.08-5.90	No significant association with asthma.	Low	
Chloramine	Lipinska-Ojrzanowska, 2017(39)	Case-series	-	-	Chloramine induced asthma in 2 cases.	Moderate	(moderate)
Chloramine: chlorine-releasing agents in combination with urine	Moore, 2017(44)	Case-series	-	-	Chlorine-releasing agents in combination with urine (chloramines) caused asthma in one case.	Moderate	
Chloramine	Walters, 2018(64)	Case-series	-	-	Chloramine caused asthma in 25 persons.	Low	
Chlorhexidine	Carder, 2019(19)	Case-series	-	-	Chlorhexidine caused asthma in 1 person	Low	
Cleaning agents (industrial)	Hoy, 2013(29)	Cohort	OR=1.1	0.8-1.7	No statistically significant association for development of self-reported asthma after 13 years of age.	Low	
Cleaning agents	Lillienberg, 2013(36)	Cohort	Men HR=2.6<br>Women=2.0	1.1-6.1<br>1.2-3.0	Statistically significant association for new-onset asthma.<br>Statistically significant association for new-onset asthma.	Low	
Cleaning agents	Lillienberg, 2014(37)	Cohort	Men HR=2.3<br>Women=2.0	1.0-5.4<br>1.2-3.1	Statistically significant association; new onset asthma (note: same cohort as for Lillienberg, 2013[23] but with another JEM).	Low	
Cleaning agents	Weinmann, 2017(65)	Cohort	Low/med=1.55<br>High=2.79	0.51-4.71<br>1.14-6.83	Dose-dependent increase in incident asthma in relation to disinfectant	Low	
Cleaning agents (LMW)	Beach, 2012(15)	CC	OR=1.16	0.91-2.21	No statistical significant increased risk after Bayesian correction for exposure misclassification; Physician billing for asthma	Low	
Cleaning agents unspecified	Walters, 2018(64)	Case-series	-	-	Chloramine caused asthma in 10 persons	Low	
Cleaning products (dilation, task)	Gonzalez, 2014(26)	CS	OR=0.81	0.39-1.65	No statistically significant association for physician-diagnosed asthma	Moderate	
Cleaning (essential tasks)	Le Moual, 2012(35)	CS	OR=1.42	0.92-2.20	No statistically significant association current asthma	Low	
Cleaning (general task)	Gonzalez, 2014(26)	CS	OR=2.26	0.95-5.35	No statistically significant association for physician-diagnosed asthma	Moderate	
Cleaning chemicals	Huntley, 2022(31)	Case-series	-	-	Cleaning chemicals cause asthma in 4 persons	Moderate	
Chemical products (cleaning)	Le Moual, 2012(35)	CS	OR=0.97	0.54-1.30	No statistically significant association for current asthma	Low	
Degreasers	Vizcaya, 2013(62)	CC	OR=1.2	0.5-3.0	No statistically significant association for clinically assessed asthma	Low	
Cleaning activities:<br><br>Internal comparison<br>- Dusting, sweeping, vacuuming	Brooks 2020(18)	CS	 <br><br>OR=0.65	 <br><br>0.34-1.26	 <br><br>Only a significant association were found for exposure to washing by machine and current asthma in the internal comparison for cleaners.	Low	

- Mopping, wet cleaning, damp wiping<br>- Cleaning the toilet			OR=1.33<br>OR=1.16	0.51-3.43<br>0.66-2.05	Comparing using an external population, all except three estimates showed a significant association.		
- Polishing, waxing			OR=0.85	0.49-1.45			
- Cleaning windows			OR=1.02	0.60-1.73			
- Cleaning kitchen			OR=0.92	0.58-1.45			
- Washing or soaking			OR=0.43	0.14-1.29			
- Washing by machine			OR=1.44	0.76-2.75			
- Cleaning machinery			OR=0.99	0.49-1.99			
External comparison							
- Dusting, sweeping, vacuuming			OR=1.72	1.09-2.71			
- Mopping, wet cleaning, damp wiping			OR=1.86	1.19-2.91			
- Cleaning the toilet			OR=1.89	1.19.2.99			
- Polishing, waxing			OR=1.60	0.86-2.95			
- Cleaning windows			OR=1.84	1.16-2.92			
- Cleaning kitchen			OR=1.77	1.08-2.89			
- Washing or soaking			OR=0.83	0.27-2.62			
- Washing by machine			OR=2.36	1.20-4.66			
- Cleaning machinery			OR=1.84	0.89-3.80			
Decalcifiers/acids	Brooks 2020(18)	CS<br>- Internal<br>- External	 OR=1.54<br>OR=2.77	 0.65-3.68<br>1.11-6.92	No significant association were found for an internal comparison with cleaners, but for comparing with an external population of service workers and bus drivers in current asthma status	Low	
Denatonium	Walters, 2018(64)	Case series	-	-	Denatonium caused asthma in one person	Low	
Dichloromethane	Carder, 2019(19)	Case-series	-	-	Dichloromethane caused asthma in 1 person	Low	
Didecyldimethylammonium (chloride)	Carder, 2019(19)	Case-series	-	-	Didecyldimethylammonium (chloride) caused asthma in 1 person	Low	
Diethanolamine	Carder, 2019(19)	Case-series	-	-	Diethanolamine caused asthma in 1 person	Low	
Disinfection (dilution, task)	Gonzalez, 2014(26)	CS	OR=4.01	1.34-12.00	Statistically significant association for physician-diagnosed asthma	Moderate	(moderate)
Disinfecting products	Ghosh, 2013(25)	Cohort	OR=1.91	1.03-3.56	Statistically significant association for adult onset asthma with airflow limitation	Low	
Disinfection products/cleaning	Dumas, 2014(21)	CS	OR=1.02	0.52-2.01	No association with asthma	Low	
Disinfection task (general task)	Gonzalez, 2014(26)	CS	OR=3.16	1.17-8.52	Statistically significant association for physician-diagnosed asthma	Moderate	
Disinfection cleaning products	Sit, 2020(54)	CC<br>Ever exp:<br>- Ref.<br>- Exposed<br>- Medium<br>- High	 <br>OR=1.00<br>OR=1.43<br>OR=1.39<br>OR=1.54<br><br>OR=1.00<br>OR=1.43	 <br>-<br>1.10-1.85<br>1.05-1.85<br>0.92-2.00<br><br>-<br>1.03-2.12	All except one exposure category were found to be significantly associated for the ever exposure, exposed the last 5 years, and current exposure groups for self-reported asthma. Sitsit<br>ss	Low	

		Exp. last 5 years:<br>- Ref.<br>- Exposed<br>- Medium<br>- High<br><br>Current exp:<br>- Ref.<br>- Exposed<br>- Medium<br>- High	OR=1.45<br>-<br>OR=1.00<br>OR=1.69<br>OR=1.66<br>-	1.03-2.04<br>-<br>-<br>1.17-2.43<br>1.13-2.42<br>-		
Disinfecting wipes	Pacheco Da Silva, 2022(46)	Cohort<br>No weekly<br>1-3 days/w<br>4-7 days/w	OR=1.00<br>OR=1.18<br>OR=1.32	-<br>1.06-1.31<br>1.13-1.55	Significant association for self-reported current asthma	Moderate
Disinfectants (weekly use)	Dumas 2020(22)	Cohort<br><br>- None<br>- Any<br>- Surface<br>- Instrument	 <br>HR=1.00<br>HR=1.12<br>HR=1.12<br>HR=1.13	 <br>-<br>0.91-1.38<br>0.87-1.43<br>0.86-1.48	No statistically association for self-reported asthma.	Low
Duration of high-level disinfectants	Dumas 2021(23)	Cohort<br><br>- <1 year<br>- 1-5 years<br>- >5 years<br><br>- ≤5 years<br>- >5 years	 <br>HR=1.00<br>HR=0.96<br>HR=1.38<br><br>HR=1.00<br>HR=1.39	 <br>-<br>0.73-1.28<br>1.03-1.85<br><br>-<br>1.04-1.86	Statistically significant association for self-reported physician diagnosed asthma with >5 years of high-level disinfectant usage.	Moderate
Duration and numbers (in the past month) of high-level disinfectants used	Dumas 2021(23)	Cohort<br><br>- ≤5 years<br>>5 years<br><br>- No current use<br>- Use of 1	 <br>HR=1.00<br>HR=1.46<br><br>HR=1.00<br>HR=1.17<br>HR=1.72	 <br>-<br>1.00-2.12<br><br>-<br>0.72-1.92<br>0.88-3.34	Statistically significant association for self-reported physician-diagnosed asthma with the usage of high-level disinfectants of >5 years and no current usage	Moderate

		- Use of ≥2				
Disinfectants (duration)	Weinmann, 2019(66)	Cohort<br>Diagnosed<br>- No exp<br>- 1-12 mo<br>- >12 mo<br><br>Current<br>- No exp<br>- 1-12 mo<br>- >12 mo	OR=1.00<br>OR=1.30<br>OR=2.99<br>OR=1.00<br>OR=1.55<br>OR=3.04	-<br>0.51-3.33<br>1.36-6.55<br>-<br>0.47-5.02<br>1.14-8.08	Significant associations were found for doctor-diagnosed asthma and current asthma for the highest exposure groups, but not for wheezing symptoms	Low
Disinfectants (per day)	Weinmann, 2019(66)	Cohort<br>Diagnosed<br>- Never<br>- <1 h/d<br>- 1-3 h/d<br>- >4 h/d<br><br>Current<br>- Never<br>- <1 h/d<br>- 1-3 h/d<br>- >4 h/d	OR=1.00<br>OR=1.27<br>OR=1.39<br>OR=1.64<br>OR=1.00<br>OR=1.22<br>OR=0.85<br>OR=1.35	-<br>0.47-3.43<br>0.45-4.34<br>0.59-4.53<br>-<br>0.38-3.91<br>0.19-3.81<br>0.40-4.60	No significant associations were found	Low
Domestic wizard	Le Moual, 2012(35)	CS	OR=0.61	0.39-0.97	Statistically significant inverse association for self-reported current asthma	Low
Drain products	Vizcaya, 2013(62)	CC	OR=0.2	0.0-2.9	No statistically significant association for clinically assessed asthma	Low
Dust mop products	Vizcaya, 2013(62)	CC	OR=1.9	0.7-5.2	No statistically significant association for clinically assessed asthma	Low
EDTA	Walters, 2018(64)	Case series	-	-	EDTA caused asthma in 1 person	Low
Enzymatic cleaners	Dumas, 2020(22)	Cohort	HR=0.97	0.72-1.30	No statistically association for self-reported asthma	Low
Enzymatic cleaners	Patel, 2020(48)	CS<br>- New onset asthma	OR=0.84	0.10-7.02	No significant association with asthma or bronchial-hyperresponsiveness symptoms	Low
Ethanolamine	Carder, 2019(19)	Case-series	-	-	Formaldehyde caused asthma in 1 person	Low
Ethanolamine containing compounds	Vandenplas, 2013(60)	Case series	-	-	An association was found in 2 persons	High
Ethylene diamine	Carder, 2019(19)	Case-series	-	-	Ethylene diamine caused asthma in 1 persons	Low
Ethylene diamine	Walters, 2018(64)	Case series	-	-	Ethylene diamine caused asthma in 1 person	Low

Endoscopy/glutaraldehyde/orthophthalaldehyde	Patel, 2020(48)	CS<br>- New onset asthma	OR=2.90	0.79-10.60	No significant association with asthma or bronchial-hyperresponsiveness symptoms	Low		
Detergents/washing powder	Brooks 2020(18)	CS<br>- Internal<br>- External	OR=1.14<br>OR=1.97	0.73-1.78<br>1.18-3.28	No significant association were found for an internal comparison with cleaners, but a significant association were found comparing with an external population of service workers and bus drivers in current asthma status	Low		
Formaldehyde	Carder, 2019(19)	Case-series	-	-	Formaldehyde caused asthma in 3 persons	Low	(moderate)	(moderate)
Formaldehyde	Dumas, 2020(22)	Cohort	HR=0.97	0.67-1.40	No statistically association for self-reported asthma	Low		
Formaldehyde	Lipinska-Ojrzanowska, 2017(39)	Case-series	-	-	Formaldehyde induced asthma in 1 case	Moderate		
Glass cleaners	Vizcaya, 2013(62)	CC	OR=1.0	0.3-2.7	No statistically significant association for clinically assessed asthma	Low		
Glass cleaning spray	Brooks, 2020(18)	CS<br>- Internal<br>- External	OR=1.07<br>OR=1.88	0.68-1.69<br>1.17-3.02	No significant association were found for an internal comparison with cleaners, but a significant association were found comparing with an external population of service workers and bus drivers in current asthma status	Low		
Glutaraldehyde	Carder, 2019(19)	Case-series	-	-	Glutaraldehyde caused asthma in 172 persons	Low	(moderate)	(moderate)
Glutaraldehyde	Dumas, 2020(22)	Cohort	HR=1.11	0.88-1.41	No statistically association for self-reported asthma	Low		
Glutaraldehyde	Dumas, 2021(23)	Cohort	HR=1.55	0.96-2.49	No statistically association for self-reported physician-diagnosed asthma	Moderate		
Glutaraldehyde	Lipinska-Ojrzanowska, 2017(39)	Case-series	-	-	Glutaraldehyde induced asthma in 1 case	Moderate		
Glutaraldehyde	Walters, 2018(64)	Case series	-	-	Glutaraldehyde caused asthma in 21 persons	Low		
Gluteraldehyde containing compounds	Vandenplas 2013(60)	Case series	-	-	An association was found in 3 persons	High		
Green products	Pacheco Da Silva, 2022(46)	Cohort<br>No weekly<br>1-3 days/w<br>4-7 days/w	OR=1.00<br>OR=1.07<br>OR=1.18	-<br>0.97-1.18<br>1.03-1.36	Significant association for self-reported current asthma	Moderate		
Home cleaning (>1 day/week)	Le Moual, 2012(35)	CS	OR=1.34	0.87-2.05	No statistically significant association for current asthma	Low		
Homemade products (cleaning)	Pacheco Da Silva, 2022(46)	Cohort<br>No weekly<br>1-3 days/w<br>4-7 days/w	OR=1.00<br>OR=1.15<br>OR=1.31	-<br>1.01-1.31<br>1.08-1.58	Significant association for self-reported current asthma	Moderate		

Hydrochloric acid	Vizcaya, 2013(62)	CC	OR=1.5	0.3-7.7	No statistically significant association for clinically assessed asthma	Low
Hydrogen peroxid	Dumas, 2020(22)	Cohort	HR=1.06	0.84-1.34	No significant association for self-reported asthma	Low
Hydrogen peroxid	Dumas 2021(23)	Cohort<br>- ≤5 years<br>- >5 years	 HR=1.00<br>HR=1.73	 -<br>0.89-3.37	No significant association for self-reported physician-diagnosed asthma	Moderate
Isothiazolinone	Walters, 2018(64)	Case-series	-	-	Isothiazolinone caused asthma in 3 persons	Low
Latex	Beach, 2012(15)	Cohort	OR=1.79	1.27-2.53	Statistically significant association; Physician billing for asthma	Low
Latex	Hoy, 2013(29)	Cohort<br>- Yes vs no<br>- Ref<br>- 1-5 years<br>- 6-15 years<br>- 16 years	 OR=1.40<br>OR=1.00<br>OR=0.72<br>OR=1.61<br>OR=2.65	 0.9-2.3	 No statistically significant association for self-reported development of asthma after 13 years of age	Low
Latex	Lillienberg, 2013(36)	Cohort	Men HR=1.7<br>Women HR= 1.3	0.7-4.2<br>0.8-2.3	No statistically significant association for new-onset asthma for both men and women	Low
Latex	Lillienberg, 2014(37)	Cohort	HR men=1.4<br>Women=1.3	0.4-4.4<br>0.9-1.8	No statistically significant association for new-onset asthma for both men and women (Note: Same cohort as Lillienberg, 2013[23] but with another JEM)	Low
Latex	Lipinska-Ojrzanowska, 2017(39)	Case-series	-	-	Latex induced asthma in three cases	Moderate
Latex	Singh, 2013(53)	CS<br>- Atopic<br>- Non-atopic	 OR=0.72<br>OR=0.78	 0.32-1.60<br>0.31-1.96	No statistically significant association for atopic and non-atopic asthma	Low
Latex antigens	Ghosh, 2013(25)		OR=1.23	0.67-2.26	No statistically significant association for adult onset asthma with airflow limitation	Low
Latex gloves	Gonzalez, 2014(26)	CS	OR=0.69	0.32-1.51	No statistically significant association for physician-diagnosed asthma	Moderate
Latex (pre- 1992)	Patel, 2020(48)	CS<br>- New onset asthma	 OR=1.24	 0.15-10.30	 No significant association with asthma or bronchial-hyperresponsiveness symptoms	Low
Latex (1992-2000)	Patel, 2020(48)	CS<br>- New onset asthma	 OR=2.43	 0.66-8.99	 No significant association with asthma but a significant association were observed in bronchial-hyperresponsiveness symptoms	Low
Limescale removers	Vizcaya, 2013(62)	CC	OR=0.2	0.1-0.7	No statistically significant association for clinically assessed asthma	Low
Liquid multi-use cleaner	Brooks 2020(18)	CS<br>- Internal	 OR=1.66<br>OR=2.49	 1.04-2.64<br>1.48-4.18	A significant association were found for an internal comparison with cleaners and for comparing with an external population of service workers and bus drivers in current asthma status	Low

		- External				
Multi-use products	Vizcaya, 2013(62)	CC	OR=2.3	0.7-7.0	No statistically significant association for clinically assessed asthma	Low
Other cleaning products (not gluraradehyde, Ortho-phtalalde, hydrogen peroxide)	Dumas, 2021(23)	Cohort	HR=1.12	0.42-3.01	No statistically association for self-reported physician-diagnosed asthma	Moderate
Other cleaning products not further specified	Brooks, 2020(18)	CS<br>- Internal<br>- External	OR=1.79<br>OR=2.00	0.91-3.35<br>1.27-3.14	Significant association was found for current asthma status	Low
Ortho-phtalaldehyde	Dumas, 2021(23)	Cohort	HR=1.20	0.44-3.22	No statistically association for self-reported physician-diagnosed asthma	Moderate
Organic solvents	Sit, 2020(54)	CC<br>Ever exp:<br>- Ref.<br>- Exposed<br>- Medium<br>- High<br>Exp. Last 5 years:<br>- Ref.<br>- Exposed<br>- Medium<br>- High<br>Current exp:<br>- Ref.<br>- Exposed<br>- Medium<br>- High	OR=1.00<br>OR=0.96<br>OR=1.10<br>-<br>OR=1.00<br>OR=0.27<br>-<br>OR=1.00<br>OR=0.82<br>OR=0.86	-<br>0.63-1.46<br>0.73-1.68<br>-<br>-<br>0.04-1.99<br>-<br>-<br>0.32-2.10<br>0.34-2.20<br>-	No significant association for self-reported asthma	Low
Peracetic acid	Carder, 2019(19)	Case-series	-	-	Peracetic acid caused asthma in 5 persons	Low
Peracetic acid	Walters, 2018(64)	Case-series	-	-	Peracetic acid caused asthma in 2 persons	Low
Perchloroethylene	Carder, 2019(19)	Case-series	-	-	Perchloroethylene acid caused asthma in 2 persons	Low
Polishes/waxes	Brooks, 2020(18)	CS<br>- Internal<br>- External	OR=1.12<br>OR=2.01	0.62-2.05<br>1.03-3.93	No significant association were found for an internal comparison with cleaners, but a significant association were found comparing with an external population of service workers and bus drivers in current asthma status	Low
Polishes and waxes	Vizcaya, 2013(62)	CC	OR=1.1	0.2-5.1	No statistically significant association for clinically assessed asthma	Low

Scented products	Pacheco Da Silva, 2022(46)	Cohort<br>No weekly<br>1-3 days/w<br>4-7 days/w	 OR=1.00<br>OR=1.14<br>OR=1.19	 -<br>1.03-1.25<br>1.06-1.33	Significant association for self-reported current asthma	Moderate		
Soaps or detergents	Vizcaya, 2013(62)	CC	OR=0.2	0.1-0.7	Soap and detergents showed a statistically significant inverse association with clinically assessed asthma	Low		
Soaking solutions preparation (task)	Gonzalez, 2014(26)	CS	OR=1.56	0.77-3.18	No statistically significant association; physician-diagnosed asthma	Moderate		
Sodium hydroxide	Walters, 2018(64)	Case series	-	-	Sodium hydroxide caused asthma	Low		
Sodium dichloroisocyanurate	Carder, 2019(19)	Case-series	-	-	Sodium dichloroisocyanurate caused asthma in 3 persons	Low		
Sodium hydroxide	Walters, 2018(64)	Case-series	-	-	Sodium hydroxide caused asthma in 1 person	Low		
Sprays	Pacheco Da Silva, 2022(46)	Cohort<br>No weekly<br>1-3 days/w<br>4-7 days/w	 OR=1.00<br>OR=1.13<br>OR=1.37	 -<br>1.02-1.24<br>1.22-1.55	Significant association for self-reported current asthma	Moderate	(moderate)	(moderate)
Spray: Solvents/stain removers	Brooks, 2020(18)	CS<br>- Internal<br>- External	 OR=1.19<br>OR=2.11	 0.47-3.02<br>0.80-5.55	No significant association were found for current asthma status	Low		
Sprays for mopping the floor	Brooks, 2020(18)	CS<br>- Internal<br>- External	 OR=1.20<br>OR=2.13	 0.71-2.04<br>1.18-3.87	No significant association were found for an internal comparison with cleaners, but a significant association were found comparing with an external population of service workers and bus drivers in current asthma status	Low		
Spray: Furniture sprays	Brooks, 2020(18)	CS<br>- Internal<br>- External	 OR=1.41<br>OR=2.50	 0.78-2.55<br>1.27-4.91	No significant association were found for an internal comparison with cleaners, but a significant association were found comparing with an external population of service workers and bus drivers in current asthma status	Low		
Spray: Sprays for carpets, rugs, or curtains	Brooks, 2020(18)	CS<br>- Internal<br>- External	 OR=3.25<br>OR=5.57	 1.16-9.10<br>1.94-16.0	A significant association were found for an internal comparison with cleaners and for comparing with an external population of service workers and bus drivers in current asthma status	Low		
Spray: Oven sprays	Brooks, 2020(18)	CS<br>- Internal<br>- External	 OR=0.66<br>OR=1.24	 0.08-5.85<br>0.14-11.0	No significant association for current asthma status	Low		
Spray: Multi-purpose antibacterial cleaning spray	Brooks, 2020(18)	CS<br>- Internal<br>- External	 OR=1.28<br>OR=2.15	 0.80-2.04<br>1.25-3.68	No significant association were found for an internal comparison with cleaners, but a significant association were found comparing with an external population of service workers and bus drivers in current asthma status	Low		
Spray: Air-refreshing spray	Brooks, 2020(18)	CS<br>- Internal<br>- External	 OR=0.97<br>OR=1.81	 0.59-1.60<br>1.02-3.23	No significant association were found for an internal comparison with cleaners, but a significant association were found comparing with an external population of service workers and bus drivers in current asthma status	Low		

Sprays	Patel, 2020(48)	CS<br>- New onset asthma	OR=1.39	0.35-5.60	No significant association with asthma or bronchial-hyperresponsiveness symptoms	Low		
Spray, mist or steam	Singh, 2013(53)	CS<br>- Atopic<br>- Non-atopic	OR=5.11<br>OR=9.57	0.52-50.23<br>0.21-42.6	No statistically significant association for both atopic and non-atopic asthma	Low		
Spray or aerosol form: Multi-use products	Vizcaya, 2013(62)	CC	OR=4.1	1.0-18.0	Statistically significant association for clinical assessed asthma	Low		
Spray or aerosol form: Degreasers	Vizcaya, 2013(62)	CC	OR=1.1	0.4-3.1	No statistically significant association for clinical assessed asthma	Low		
Spray or aerosol form: Dust mop products	Vizcaya, 2013(62)	CC	OR=1.5	0.6-3.9	No statistically significant association for clinical assessed asthma	Low		
Spray or aerosol form: Spray: Limescale removers	Vizcaya, 2013(62)	CC	OR=1.5	0.5-5.0	No statistically significant association for clinical assessed asthma	Low		
Spray or aerosol form: Glass cleaners	Vizcaya, 2013(62)	CC	OR=1.2	0.3-5.9	No statistically significant association for clinical assessed asthma	Low		
Spray: Number of different sprays	Vizcaya, 2013(62)	CC<br>- 0<br>- 1-2<br>- 3-5	OR=1.0<br>OR=0.8<br>OR=2.1	-<br>0.3-2.4<br>0.6-7.4	No statistically significant association for clinical assessed asthma	Low		
Spray use	Weinmann, 2017(65)	Cohort	OR: Low=0.70<br>Medium=0.78<br>High=2.79	0.23-2.06<br>0.26-2.36<br>0.84-9.20	Weak indication of a dose-dependent increase in incident asthma in relation to spray use	Low		
Spray use at work (task)	Gonzalez, 2014(26)	CS	OR=0.84	0.42-1.69	No statistically significant association for physician-diagnosed asthma	Moderate		
Spray use (1 type)	Le Moual, 2012(35)	CS	OR=0.68	0.44-1.04	No statistically significant association for current asthma	Low		
Spray use (2 types)	Le Moual, 2012(35)	CS	OR=1.67	1.08-2.56	Statistically significant association for current asthma	Low		
Stain removers	Vizcaya, 2013(62)	CC	OR=0.8	0.1-4.1	No statistically significant association for clinically assessed asthma	Low		
Triclosan	Walters, 2018(64)	Case-series	-	-	Triclosan caused asthma in 1 person	Low		
Quats	Dumas, 2020	Cohort	HR=1.00	0.79-1.26	No statistically association for self-reported asthma	Low		
Quaternary ammonia compounds (QAC)	Migueres, 2021(43)	Cohort			22 subjects diagnosed with QUC-induced asthma	Low	(moderate)	(moderate)
Quaternary ammonia compounds (QAC)	Vandenplas, 2013(60)	Case series	-	-	An association was found in 10 persons	High		
Quaternary ammonia compounds (QAC)	Gonzalez, 2014(26)	CS	OR=7.56	1.84-31.05	Statistically significant association for physician-diagnosed asthma	Moderate		
QAC and glutaraldehyde	Vandenplas 2013(60)	Case series	-	-	An association was found in 1 persons	High		
QAC	Walters, 2018(64)	Case-series	-	-	Quaternary ammonium compounds caused asthma in 9 persons	Low		
Xylenone	Carder, 2019(19)	Case-series	-	-	Xylenone caused asthma in one person	Low		

1,1,1-trichloroethane	Carder, 2019(19)	Case-series	-	-	1,1,1-trichloroethane caused asthma in one person	Low		
**Hydrocarbons (oil combustion byproducts)**								
Benzene	Lawrence 2022(34)	Cohort			Significant association for development of self-reported asthma with a significant exposure-response relation.	Moderate		
		- Quartile 1	RR= 1.00	-				
		- Quartile 2	RR=1.60	1.30-1.98				
		- Quartile 3	RR=1.91	1.55-2.35				
		- Quartile 4	RR=2.67	2.17-3.28				
Ethylbenzene	Lawrence 2022(34)	Cohort			Significant association for development of self-reported asthma with a significant exposure-response relation.	Moderate		
		- Quartile 1	RR=1.00	-				
		- Quartile 2	RR=1.55	1.26-1.91				
		- Quartile 3	RR=1.91	1.56-2.34				
		- Quartile 4	RR=2.66	2.16-3.27				
Toluene	Lawrence 2022(34)	Cohort			Significant association for development of self-reported asthma with a significant exposure-response relation.	Moderate		
		- Quartile 1	RR=1.00	-				
		- Quartile 2	RR=1.49	1.21-1.84				
		- Quartile 3	RR=1.80	1.46-2.23				
		- Quartile 4	RR=2.99	2.44-3.66				
Total hydrocarbons	Lawrence 2022(34)	Cohort			Significant association for development of self-reported asthma with a significant exposure-response relation.	Moderate		
		- Quintile 1	RR=1.00	-				
		- Quintile 2	RR=1.50	1.17-1.92				
		- Quintile 3	RR=1.76	1.38-2.25				
		- Quintile 4	RR=2.15	1.70-2.72				
		- Quintile 5	RR=2.95	2.33-3.74				
n-Hexane	Lawrence 2022(34)	Cohort			Significant association for development of self-reported asthma with a significant exposure-response relation.	Moderate		
		- Quartile 1	RR=1.00	-				
		- Quartile 2	RR=1.66	1.34-2.05				
		- Quartile 3	RR=2.08	1.69-2.56				
		- Quartile 4	RR=2.71	2.19-3.37				
Xylenes	Lawrence 2022(34)	Cohort			Significant association for development of self-reported asthma with a significant exposure-response relation.	Moderate		
		- Quartile 1	RR=1.00	-				
		- Quartile 2	RR=1.25	1.02-1.53				
		- Quartile 3	RR=1.56	1.28-1.90				
		- Quartile 4	RR=2.62	2.16-3.17				
**Highly reactive chemicals**							Limited or contradictory evidence	Limited or contradictory evidence
Highly reactive chemicals	Hoy, 2013(29)	Cohort	OR=1.2	0.7-2.0	No statistically significant association for development of self-reported asthma after 13 years of age	Low		
Highly reactive chemicals	Ghosh, 2013(25)	Cohort	OR=1.23	0.61-4.28	No statistically significant association for adult onset asthma with airflow limitation	Low		
Highly reactive chemicals	Dumas, 2014(21)	CS	OR=0.97	0.61-1.53	No statistically significant association for physician- diagnosed asthma	Low		
Reactive chemical	Lillienberg, 2013(36)	Cohort	Men HR=1.0<br>Women=1.6	0.3-3.1<br>0.8-3.0	No statistically significant association for new-onset asthma<br>No statistically significant association for new-onset asthma	Low		
Highly reactive chemicals	Lillienberg, 2014(37)	Cohort	Men HR=2.1<br>Women=1.4	0.99-4.30<br>0.96-2.00	No statistically association for new-onset asthma (note: same cohort as for Lillienberg, 2013[23] but with another JEM)	Low		
Reactive chemicals-isocyanates	Ghosh, 2013(25)	Cohort	OR=1.91	0.63-5.79	No statistically significant association for adult onset asthma with airflow limitation	Low		

**Unspecified group of other chemicals**							Limited or contradictory evidence	Moderate evidence
Acrylates	Lillienberg, 2013(36)	Cohort	HR=1.8	0.8-3.7	No statistically significant association for new-onset asthma	Low		(moderate)
Acrylates	Suojalehto, 2020(68)	Case-reports	-	-	Acrylate-induced asthma ascertained by SIC in 55 cases	High		
Acrylates	Walters, 2017(63)	Case-series	-	-	Acrylates caused asthma in two patients	Low		
Acrylic polymers	Walters, 2017(63)	Case-series	-	-	Acrylic polymers caused asthma in 2 patients	Low		
Cyanoacrylates including alkyl-cyanoacrylates	Walters, 2017(63)	Case-series	-	-	Cyanoacrylates caused asthma in 8 patients	Low		
Chlorhexidine	Wittczak, 2013(67)	Case-series	-	-	Chlorhexidine caused asthma in 2 patients	High		
Epoxy	Lillienberg, 2013(36)	Cohort	HR=2.4	1.3-4.5	Statistically significant association for new-onset asthma	Low		(moderate)
Epoxy components: epoxy resin	Suojalehto, 2019(57)	Case-series	-	-	Epoxy resin caused sensitizer-induced occupational asthma in some exposed cases	Moderate		
Epoxy components: triglycidyl iso-cyanurate)	Suojalehto, 2019(57)	Case-series	-	-	Triglycidyl isocyanurate caused sensitizer-induced occupational asthma in some exposed cases	Moderate		
Epoxy resin, sand and phthalic anhydride countertops fabrication	Tustin, 2021(59)	CS			7 workers with confirmed or suspected work-related asthma working with fabrication of artificial stone countertop	Moderate		
Epoxy resin	Migueres, 2021(43)	Case-series	-	-	Epoxy resin caused asthma in 7 persons	Low		
Hair dyes e.g., persulfates, permanent wave solutions, hair bleach	Helaskoski, 2014(27)	Case-series	-	-	Association found in 5 cases	Moderate		
Methyl methacrylates	Walters, 2017(63)	Case-series	-	-	Methyl methacrylates caused asthma in 8 patients	Low		
Potassium aluminium tetrafluoride	Lastovkova, 2015(33)	Case-series	-	-	4 of 5 patients had occupational asthma based	Moderate		
Polyfunctional aziridine	Mason, 2020(42)	Case-report	-	-	Asthma due to polyfunctional aziridine in one patient	High		
Polyvinyl Chloride (PVC)	Song, 2013(55)	Case-report	-	-	Polyvinyl chloride (and nickel) caused asthma in 1 patient	Moderate		

Persulfate salts (hair-bleaching products)	Hougaard, 2012(28)	Case-report	-	-	An association between persulfate salts and asthma was found in 1 case	Low
3-(Bromomethyl)-2-chloro-4-(methylsulfonyl)-benzoic acid, BCMBA	Suojalehto, 2018(56)	Case series	-	-	An association between 3-(Bromomethyl)-2-chloro-4-(methylsulfonyl)-benzoic and asthma was found	High

BHR; Bronchial hyperresponsiveness, CC; Case-control study, CS; Cross-sectional study, HR; Hazard ratio, mo: Months, vs; Versus, OR; Odds ratio, POR; Prevalence odds ratio, RR; Relative risk
